# Associations and prognostic implications of myocardial tissue injury stages in ST-elevation myocardial infarction using the Canadian Cardiovascular Society classification

**DOI:** 10.1093/ehjci/jeaf250

**Published:** 2025-08-22

**Authors:** Ivan Lechner, Jaclyn Carberry, Thomas Stiermaier, Christina Tiller, Magdalena Holzknecht, Fritz Oberhollenzer, Alex Kaser, Johannes Mair, Agnes Mayr, Hans-Josef Feistritzer, David Carrick, Clemens Dlaska, Axel Bauer, Holger Thiele, Ingo Eitel, Bernhard Metzler, Colin Berry, Martin Reindl, Sebastian J Reinstadler

**Affiliations:** University Clinic of Internal Medicine III, Cardiology and Angiology, Medical University of Innsbruck, Anichstr. 35, Innsbruck 6020, Austria; British Heart Foundation Glasgow Cardiovascular Research Centre, School of Cardiovascular and Metabolic Health, University of Glasgow, Glasgow, UK; University Heart Centre Lübeck, Medical Clinic II (Cardiology/Angiology/Intensive Care Medicine), University Hospital Schleswig-Holstein, Lübeck, Germany; German Centre for Cardiovascular Research (DZHK), Partner Site Hamburg/Kiel/Lübeck, Lübeck, Germany; University Clinic of Internal Medicine III, Cardiology and Angiology, Medical University of Innsbruck, Anichstr. 35, Innsbruck 6020, Austria; University Clinic of Internal Medicine III, Cardiology and Angiology, Medical University of Innsbruck, Anichstr. 35, Innsbruck 6020, Austria; University Clinic of Internal Medicine III, Cardiology and Angiology, Medical University of Innsbruck, Anichstr. 35, Innsbruck 6020, Austria; University Clinic of Internal Medicine III, Cardiology and Angiology, Medical University of Innsbruck, Anichstr. 35, Innsbruck 6020, Austria; University Clinic of Internal Medicine III, Cardiology and Angiology, Medical University of Innsbruck, Anichstr. 35, Innsbruck 6020, Austria; University Clinic of Radiology, Medical University of Innsbruck, Innsbruck, Austria; Department of Internal Medicine/Cardiology, Heart Centre Leipzig at Leipzig University and Leipzig Heart Science, Leipzig, Germany; British Heart Foundation Glasgow Cardiovascular Research Centre, School of Cardiovascular and Metabolic Health, University of Glasgow, Glasgow, UK; Department of Cardiology, University Hospital Hairmyres, East Kilbride, UK; University Clinic of Internal Medicine III, Cardiology and Angiology, Medical University of Innsbruck, Anichstr. 35, Innsbruck 6020, Austria; Digital Cardiology Lab, Medical University of Innsbruck, Innsbruck, Austria; University Clinic of Internal Medicine III, Cardiology and Angiology, Medical University of Innsbruck, Anichstr. 35, Innsbruck 6020, Austria; Department of Internal Medicine/Cardiology, Heart Centre Leipzig at Leipzig University and Leipzig Heart Science, Leipzig, Germany; University Heart Centre Lübeck, Medical Clinic II (Cardiology/Angiology/Intensive Care Medicine), University Hospital Schleswig-Holstein, Lübeck, Germany; German Centre for Cardiovascular Research (DZHK), Partner Site Hamburg/Kiel/Lübeck, Lübeck, Germany; University Clinic of Internal Medicine III, Cardiology and Angiology, Medical University of Innsbruck, Anichstr. 35, Innsbruck 6020, Austria; British Heart Foundation Glasgow Cardiovascular Research Centre, School of Cardiovascular and Metabolic Health, University of Glasgow, Glasgow, UK; University Clinic of Internal Medicine III, Cardiology and Angiology, Medical University of Innsbruck, Anichstr. 35, Innsbruck 6020, Austria; University Clinic of Internal Medicine III, Cardiology and Angiology, Medical University of Innsbruck, Anichstr. 35, Innsbruck 6020, Austria

**Keywords:** ST-segment elevation myocardial infarction, cardiac magnetic resonance, microvascular injury, prognosis

## Abstract

**Aims:**

The recently proposed Canadian Cardiovascular Society (CCS) classification categorizes post-infarction tissue injury into four stages, potentially improving risk stratification and guiding cardioprotective strategies. Its clinical and prognostic relevance in ST-elevation myocardial infarction (STEMI) remains unclear. We aimed to compare clinical characteristics across CCS stages and validate their prognostic implications in STEMI.

**Methods and results:**

We analysed 1109 STEMI patients included in three prospective studies. Cardiac magnetic resonance (CMR) imaging was performed 3 (interquartile range 2–5) days after myocardial infarction (MI) and patients were classified as follows: Stage 1 (aborted MI), Stage 2 (MI with necrosis and absence of microvascular injury), Stage 3 (MI with necrosis and microvascular obstruction), and Stage 4 (MI with necrosis and intramyocardial haemorrhage). This analysis revealed distinct patterns of clinical presentation, biomarker profiles, and cardiac function across the different CCS stages. There were differences in adverse clinical event rates and mortality between CCS stages [major adverse cardiovascular event (MACE): 0.7%, 3.4%, 3.1%, and 15.7%, *P* < 0.001 and mortality: 0.7%, 1.7%, 0.9%, and 6.3%, *P* < 0.001]. The CCS stage had a moderate to good predictive value for MACE and mortality [area under the curve (AUC) 0.74 (95% CI: 0.68–0.80), *P* < 0.001 and AUC 0.71 (95% CI: 0.61–0.80), *P* < 0.001] at 12 months, respectively. CCS stages were independently associated with MACE in multivariable Cox regression [hazard ratio: 2.18 (95% CI: 1.70–2.78), *P* < 0.001].

**Conclusion:**

This study describes clinical characteristics across CCS stages and provides insights into their prognostic implications in a large cohort of STEMI patients reperfused by percutaneous coronary intervention. These data should inform the use of CCS stages in future trial designs.

## Graphical Abstract

**Structured Graphical Abstract:**
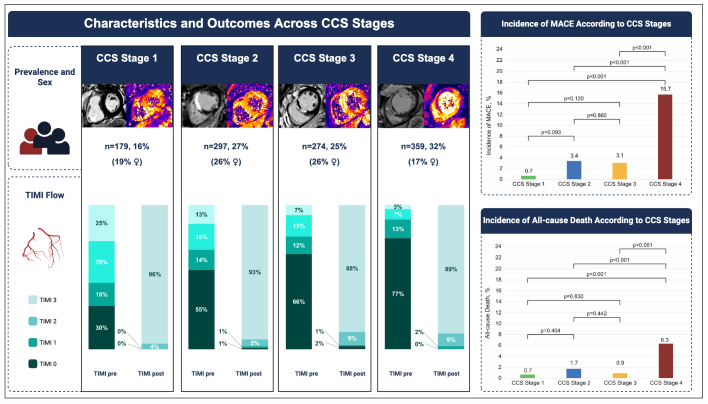
The graphical abstract summarizes key elements of the study, including the prevalence, sex distribution, pre- and post-interventional thrombolysis in myocardial infarction (TIMI) flow grades, representative cardiac magnetic resonance (CMR) images, and 12-month outcomes across Canadian Cardiovascular Society (CCS) stages 1–4 in patients with ST-elevation myocardial infarction (STEMI). Created with BioRender.

**See the editorial comment for this article ‘Tissue-level classification of STEMI—outclassed in the first round?’, by S. Plein, https://doi.org/10.1093/ehjci/jeaf374.**

## Introduction

The management of patients with ST-elevation myocardial infarction (STEMI) has advanced significantly in recent decades, resulting in a substantial reduction in cardiovascular mortality.^1–3^ Nevertheless, there is still a significant risk of adverse cardiovascular outcomes, including long-term adverse remodelling and associated heart failure.^4^ This residual risk is primarily determined by the severity of myocardial tissue damage.^1,5–11^ Infarct severity is highly dependent on prompt reperfusion, which is a key to salvage the affected ‘myocardium at risk’.^12^ However, restoring epicardial blood flow through percutaneous coronary intervention (PCI) may also trigger a cascade of deleterious pathophysiological processes (e.g. inflammation, endothelial dysfunction, and disruption)—collectively termed myocardial ischaemia–reperfusion injury—that can further exacerbate myocardial tissue damage.^5,10,11,13–15^ Importantly, the extent of infarcted tissue (infarct size) is not the sole determinant of outcome following STEMI. Other characteristics of infarct pathology, such as microvascular obstruction (MVO) and intramyocardial haemorrhage (IMH), are increasingly recognized as tissue biomarkers with strong prognostic implications.^7,10^ Therefore, a detailed understanding of the nuances of myocardial tissue injury has the potential to become a key tool in assessing and managing the residual risk post-myocardial infarction (MI).

Recent efforts to classify the heterogeneous nature of infarct severity have led to the development of the Canadian Cardiovascular Society (CCS) classification of acute atherothrombotic MI.^16,17^ This novel staging scheme, based on the severity of tissue injury as assessed by cardiac magnetic resonance (CMR) imaging, divides MI into four progressively more severe stages of tissue injury, offering a granular perspective on the pathophysiology of acute MI.^16^ The classification ranges from Stage 1, characterized by primarily reversible injury with myocardial oedema but minimal or no cardiomyocyte necrosis, to Stage 4, which involves microvascular destruction leading to IMH in addition to cardiomyocyte necrosis and MVO.^17^ The authors describe their document as a starting point for further clinical and scientific refinement, with the goal of improving MI-related outcomes. To date, no study has specifically investigated the prevalence, clinical characteristics, and prognostic implications of the proposed CCS classification scheme. Therefore, the objectives of our study were to: (i) describe the prevalence of the four stages of myocardial tissue damage; (ii) compare clinical characteristics, including biomarker release patterns; and (iii) evaluate the prognostic usefulness in predicting major adverse cardiovascular events (MACE; hospitalization for heart failure and mortality) at 1 year in patients with STEMI treated by PCI who had undergone CMR at a standardized time point early after the index event.

## Methods

### Study design and population

This multicentric, individual patient data analysis included STEMI patients from three prospective CMR cohort studies conducted in Austria [MARINA-STEMI (Magnetic Resonance Imaging In Acute ST-Elevation Myocardial Infarction); NCT04113356], Scotland [BHF MR-MI; British Heart Foundation magnetic resonance-myocardial infarction (Detection and Significance of Heart Injury in STEMI); NCT02072850], and sites in Germany and Austria [HEM-CMR (Haemorrhage Assessed by Cardiac Magnetic Resonance in STEMI)]. The detailed study protocols have been previously published,^10,18,19^ and the individual inclusion and exclusion criteria of the studies are provided in the Supplementary data online, *Table S1*.

Patients were categorized into four groups (CCS stages 1–4) based on CMR tissue characteristics and as suggested by the CCS classification.^17^ CCS stage 1 is defined by the presence of myocardial oedema and subendocardial injury of <5% of left ventricular myocardial mass (LVMM).^17,20^ CCS stage 2 is defined as myocardial injury ≥5% of LVMM but without the presence of microvascular injury. CCS stage 3 is defined by the presence of microvascular injury, as defined by the presence of MVO and the absence of IMH. Finally, CCS stage 4 is defined when microvascular injury leading to IMH is present.

Since the CCS stage 1 definition is not uniform,^17^ we have evaluated the following additional definitions: (a) absence of any focal late gadolinium enhancement (LGE) or (b) minimal myocardial injury defined as <1% of LGE of LVMM. Details for these CCS stage 1 classifications are shown in the supplementary section (see Supplementary data online, *Tables S3* and *S4*, respectively).

Killip classification and thrombolysis in myocardial infarction (TIMI) risk score was assessed for each patient using admission parameters.^21^ Additionally, the Global Registry of Acute Coronary Events (GRACE) 2.0 score^22^ was calculated in a subset of the cohort with complete data availability (*n* = 864).

We evaluated the clinical endpoint of MACE, defined as all-cause death or new congestive heart failure at 12 months. In addition, all-cause mortality assessed at 12 months was analysed as further clinical endpoint.

The detailed definitions of endpoints in each study have been published previously.^10,23,24^ In patients with more than one event during follow-up, only the first endpoint was used for the composite MACE endpoint. For all-cause mortality, deaths were included regardless of whether the event was the first or subsequent event.

All studies were approved by the responsible research ethics committees and were conducted in compliance with the Declaration of Helsinki. Every patient included gave written informed consent.

### Biomarker measurements

Biomarker analyses were limited to the MARINA-STEMI study cohort (CCS 1: *n* = 95, 16%, CCS 2: *n* = 160, 27%, CCS 3: *n* = 147, 24%, and CCS 4: *n* = 198, 33%), as standardized and comprehensive biomarker analyses were available only in this study (*n* = 600). In MARINA-STEMI, blood samples were obtained by peripheral vein puncture on hospital admission and sequentially 6 ± 2, 12 ± 6, 24 ± 6, 48 ± 6, and 72 ± 6 h after primary PCI. The median number of blood values per patient was 30 [interquartile range (IQR) 28–30]. The peak value was defined as the highest value recorded from time of admission up to 72 h post-PCI. Time to peak was calculated as the hours from PCI to the highest recorded value. All samples were analysed as part of routine laboratory testing at the Central Laboratory of the Medical University of Innsbruck by personnel blinded to the study data. Biomarker assessments for high-sensitivity cardiac troponin T (hs-cTnT) were performed using an enzyme immunoassay (hs-cTnT; E 170, Roche Diagnostics^®^, Vienna, Austria). N-terminal pro-B-type natriuretic peptide (NT-pro-BNP) levels were determined using a commercially available assay (E170 instrument proBNP II assay, Roche Diagnostics^®^, Vienna, Austria).^25^ Concentrations of creatine kinase (CK) and serum creatinine were determined by using a standard enzymatic assay (Roche Diagnostics^®^) as previously described.^26^ Acute kidney injury (AKI) was defined as an absolute serum creatinine increase of ≥0.3 mg/dL within 48 h post-PCI compared with admission serum creatinine.^27^ High-sensitive C-reactive protein (hs-CRP) analyses were performed using the c702 Cobas^®^ 8000 Modular Analyzer (Roche Diagnostics^®^).^28^

### Cardiac magnetic resonance

Details on image acquisition and post-processing of the three CMR studies have been described previously.^6,10,19^ In brief, left ventricular (LV) function and volumes were assessed by steady-state free precession technique. LGE images were obtained 10–15 min after application of 0.15–0.2 mmol/kg gadolinium-based contrast agents. Infarct area was defined by ‘hyper-enhancement’ at a threshold of +5 SD above the signal intensity of remote myocardial tissues of the opposite LV. Infarct size was expressed as percentage of LVMM. MVO was defined by ‘hypo-enhancement’ within the infarct area on LGE images.

IMH was assessed by T2* mapping acquired in three short-axis slices (basal, mid, and apical) before administration of the contrast agent. A side-by-side visual comparison between the LGE images and T2* map was used to localize the infarct region on the corresponding T2* mapping image. IMH was defined as a region of hypointense core within the infarcted area with T2* reduction <20 ms.^10^

All post-processing analyses were performed by experienced readers who were blinded to all other clinical data, using dedicated software platforms: Syngo.via (Siemens Healthcare, Erlangen, Germany), CVi42 (Circle Cardiovascular Imaging Inc., Calgary, Alberta, Canada), and IMPAX (Agfa Healthcare, Bonn, Germany).

### Statistical analyses

Statistical analysis was performed with SPSS Statistics 29.0.1 (IBM, Armonk, NY, USA) and MedCalc Version 20.022 (Ostend, Belgium). The distribution of data was tested using the Shapiro–Wilk test. Categorical variables are depicted as frequencies with corresponding percentages. Continuous variables are presented as mean ± SD or median with IQR, according to their distribution. Differences between two groups were tested using Student’s *t*-test, Mann–Whitney *U*-test, or χ^2^ test, as appropriate; Kruskal–Wallis test or χ^2^ test were used to test differences between more than two groups, as appropriate.

Differences in trends were evaluated by means of the Jonckheere–Terpstra trend test for continuous variables and linear-by-linear association for categorical variables. MACE endpoint percentages were calculated for 1 year. One-year follow-up analyses were performed by censoring patients who either had shorter follow-up without an event or who had an event after the 1-year follow-up period. Receiver operating characteristic (ROC) analyses were used to quantify the discriminative power of MACE predictors at 1 year. Area under the curves (AUCs) were compared using the nonparametric method developed by DeLong *et al*.^29^ Following Rice and Harris, AUC values were categorized as negligible (≤0.55), small (0.56–0.63), moderate (0.64–0.70), and strong (≥0.71).^30^

Multivariable stepwise Cox proportional hazards regression was performed to assess the prognostic significance of CCS stages for MACE. Cox analyses were not restricted to a fixed follow-up time point and incorporated all available event data regardless of follow-up duration. Variables considered for inclusion in the model were derived from *Table 1*, encompassing patient characteristics, angiographic parameters, global cardiac function parameters, and CCS stages. The inclusion and exclusion criteria for the model were *P* < 0.05 and *P* > 0.10, respectively. To avoid multicollinearity, composite risk scores such as the TIMI risk score and GRACE score were not included in the main model; instead, their individual components were assessed separately. An additional analysis including the TIMI and GRACE scores is provided in the Supplementary Data. To allow for direct comparison of hazard ratios (HRs) with different scales, all HRs are presented for 1 SD increase.

**Table 1:** Baseline characteristics according to CCS stages

	Total population (*n* = 1109)	CCS 1 (*n* = 179, 16%)	CCS 2 (*n* = 297, 27%)	CCS 3 (*n* = 274, 25%)	CCS 4 (*n* = 359, 32%)	*P*-value
Patient characteristics						
Age, years	58 [51–68]	59 [53–68]	58 [51–69]	58 [51–68]	58 (52–68)	0.851
Female sex, *n* (%)	242 (22)	34 (19)	77 (26)	70 (26)	61 (17)	**0.012**
Body mass index, kg/m^2^	27 [25–29]	26 [25–30]	27 [25–30]	26 [25–29]	27 [25–29]	0.509
Hypertension, *n* (%)	503 (45)	77 (43)	130 (44)	138 (50)	158 (44)	0.292
Hyperlipidaemia, *n* (%)	476 (43)	82 (46)	141 (48)	108 (40)	145 (40)	0.150
Diabetes mellitus, *n* (%)	117 (11)	21 (12)	29 (10)	26 (10)	41 (11)	0.789
Smoker, *n* (%)	608 (55)	103 (58)	162 (55)	138 (51)	205 (58)	0.339
TIMI-risk score	3 [2–5]	3 [1–4]	3 [1–5]	3 [2–5]	3 [2–5]	**0.001**
GRACE 2.0 score^b^	108 [93–125]	107 [93–121]	104 [89–123]	107 [93–123]	111 [94–129]	0.064
Admission Killip-class						**<0.001**
1	793 (72)	154 (86)	222 (75)	203 (74)	214 (60)	
2	281 (25)	23 (13)	72 (24)	67 (25)	119 (33)	
3	26 (2)	1 (<1)	3 (1)	3 (1)	19 (5)	
4	9 (1)	1 (<1)	0 (0)	1 (<1)	7 (2)	
AKI, *n* (%)	27 (2.4)	5 (2.8)	5 (1.7)	8 (2.9)	9 (2.5)	0.768
Total ischaemic time, min	179 [114–311]	140 [96–240]	185 [122–334]	182 [115–310]	200 [121–337]	**<0.001**
Biomarkers characteristics^a^						
Hs-cTnT, peak, ng/L	4776 [2155–8076]	902 [365–1830]	3265 [1870–5281]	5402 [3466–7533]	8198 [5199–13 816]	
CK, peak, U/L	1913 [980–3508]	454 [242–862]	1410 [915–2104]	2284 [1482–3345]	3614 [2237–4736]	**<0.001**
NT-pro-BNP, peak, ng/L	1453 [758–2716]	778 [410–1661]	1265 [719–2156]	1470 [838–3219]	2107 [1179–3651]	**<0.001**
Hs-CRP, peak, mg/dL	2.7 [1.3–4.9]	1.6 [0.6–2.8]	1.9 [1.1–3.4]	3.3 [1.6–5.3]	4.2 [2.2–6.8]	**<0.001**
Angiographic characteristics						
Culprit lesion, *n* (%)						**<0.001**
RCA	449 (41)	95 (53)	138 (47)	115 (42)	101 (28)	
LAD	478 (43)	63 (35)	113 (38)	124 (45)	178 (50)	
LCX	173 (16)	17 (10)	45 (15)	34 (12)	77 (21)	
RI	4 (<1)	0 (0)	0 (0)	1 (<1)	3 (1)	
LM	5 (<1)	4 (2)	1 (<1)	—	—	
Vessel disease, *n* (%)						0.544
Single-vessel	619 (56)	98 (55)	174 (59)	156 (57)	191 (53)	
Multi-vessel	490 (44)	81 (45)	123 (41)	118 (43)	168 (47)	
TIMI-flow 0 pre-PCI, *n* (%)	657 (61)	51 (30)	155 (55)	175 (66)	276 (77)	**<0.001**
TIMI-flow 3 post-PCI, *n* (%)	976 (91)	162 (96)	264 (93)	232 (88)	318 (89)	**0.005**
CMR characteristics						
Infarct size, % LVMM	16 [8–26]	2 [0–3]	12 [8–18]	19 [14–26]	27 [18–36]	**<0.001**
LVEF, %	50 [43–57]	57 [52–62]	52 [47–58]	50 [42–55]	45 [39–52]	**<0.001**
LVEDVi, mL/m^2^	80 [70–92]	74 [63–84]	78 [68–89]	80 [70–91]	85 [75–96]	**<0.001**
LVESVi, mL/m^2^	40 [31–49]	31 [25–38]	37 [30–46]	41 [32–50]	45 [38–55]	**<0.001**
Clinical outcomes						
MACE	63 (7.0)	1 (0.7)	8 (3.4)	7 (3.1)	47 (15.7)	**<0.001**
All-cause death	26 (2.9)	1 (0.7)	4 (1.7)	2 (0.9)	19 (6.3)	**<0.001**

Bold values indicate statistical significance defined as *P* < 0.05.

CCS, Canadian Cardiovascular Society; TIMI, thrombolysis in myocardial infarction; GRACE, Global Registry of Acute Coronary Events; hs-cTnT, high-sensitivity cardiac troponin T; CK, creatine kinase; NT-pro-BNP, N-terminal pro-B-type natriuretic peptide; hs-CRP, high-sensitive C-reactive protein; RCA, right coronary artery; LAD, left anterior descending; LCX, left circumflex artery; RI, ramus intermedius; LM, left main; PCI, percutaneous coronary intervention; CMR, cardiac magnetic resonance; LVMM, left ventricular myocardial mass; LVEF, left ventricular ejection fraction; MACE, major adverse cardiovascular event.

^a^Data available for patients in the MARINA-STEMI study (*n* = 600).

^b^Data available for *n* = 864 patients.

MACE-free survival was depicted by Kaplan–Meier graphs and differences between groups were calculated by log-rank test. A two-tailed *P*-value of <0.05 was considered statistically significant.

## Results

### Study population

A total of 1109 STEMI patients (22% were female), treated by PCI at a median age of 58 (IQR 51–68) years, comprised the final study population.

Patients were enrolled across three prospective CMR studies: HEM-CMR (2009–2016), BHF MR-MI (2011–2012), and MARINA-STEMI (2015–2022). Baseline demographics, angiographic characteristics, biomarker characteristics, and CMR parameters, according to CCS stages (CCS 1–4), are provided in *Table 1*.

### Prevalence and baseline characteristics of CCS stages

**Figure 1:**
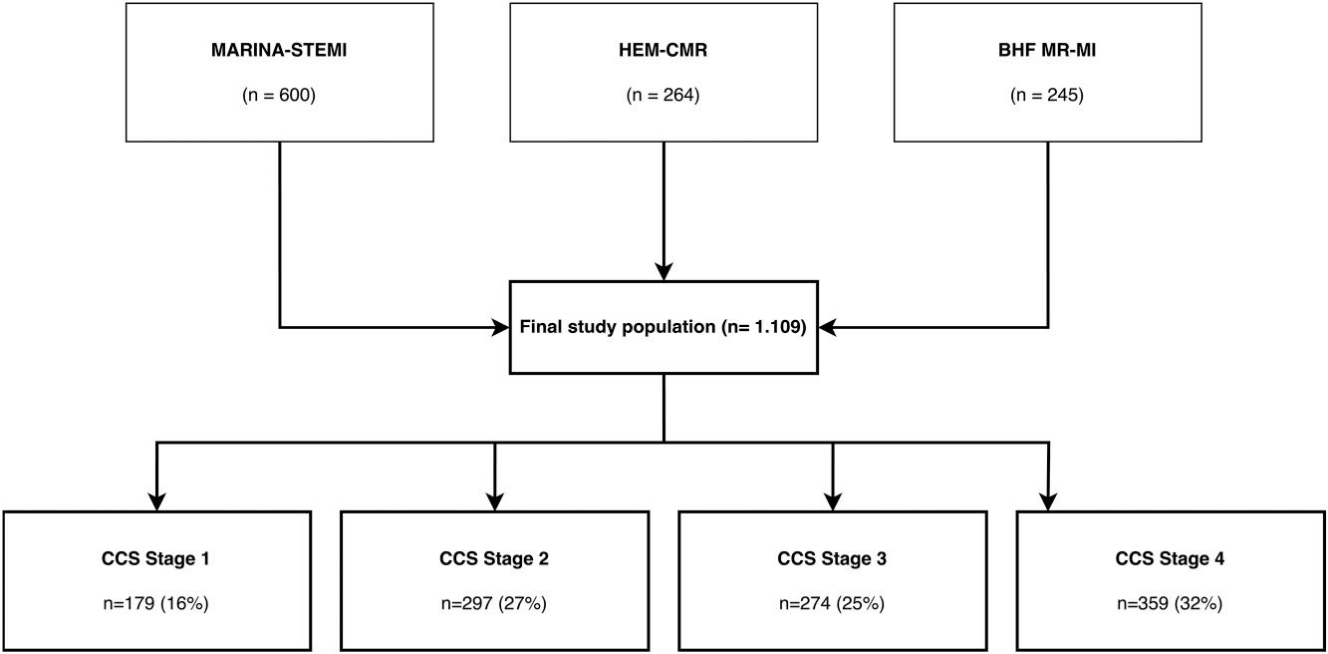
Study population and prevalence of CCS stages. The figure provides a visual overview of the pooled cohort and the distribution of patients according to the CCS classification of myocardial tissue injury (Stages 1–4). MARINA-STEMI, Magnetic Resonance Imaging In Acute ST-Elevation Myocardial Infarction; CMR, cardiac magnetic resonance; BHF, British Heart Foundation; MR, magnetic resonance; MI, myocardial infarction; HEM-CMR, Haemorrhage Assessed by Cardiac Magnetic Resonance in STEMI.

CCS stage 1 occurred in 179 (16%) patients. CCS stage 2 occurred in 297 (27%) patients, and CCS stages 3 and 4 occurred in 274 (25%) and 359 (32%) patients, respectively (Graphical Abstract, *Figure 1*). Female patients represented a higher proportion in CCS 2 (26%) and 3 (26%), as compared with CCS 1 (19%) and 4 (17%) (between groups difference, *P* = 0.012). Prevalence and baseline characteristics of CCS stages by alternative definitions are presented in Supplementary data online, *Tables S3* and *S4*, respectively.

The median total ischaemic time was 179 (IQR 114–311) minutes, with differences between CCS stages (*P* < 0.001). Differences in total ischaemic time were found between CCS 1 and CCS 2 (*P* < 0.001), CCS 1 and CCS 3 (*P* = 0.001), and CCS 1 and CCS 4 (*P* < 0.001). However, there were no differences in total ischaemic time between CCS 2 and CCS 3 (*P* = 0.407), CCS 3 and CCS 4 (*P* = 0.116), or CCS 2 and CCS 4 (*P* = 0.448), respectively.

Median TIMI-risk score of the study cohort was 3 [IQR 2–5]. There was an overall increase in TIMI-risk score across CCS stages (*P* < 0.001 for trend), with the lowest score observed in CCS 1 (3 [IQR 1–4]) and the highest in CCS 4 (3 [2–5], *P* = 0.001).

Pairwise comparison showed no significant differences in TIMI-risk scores between CCS 1 and CCS 2 (*P* = 0.988), CCS 1 and CCS 3 (*P* = 0.235), or CCS 2 and CCS 3 (*P* = 0.207). However, TIMI-risk scores in CCS 4 were significantly higher than in CCS 1 (*P* = 0.002), CCS 2 (*P* < 0.001), or CCS 3 (*P* = 0.003).

Median GRACE 2.0 score for the study cohort was 108 (IQR 93–125). Although there was an overall increase in GRACE 2.0 score across CCS stages (*P* = 0.019 for trend), no statistically significant differences between groups were observed (*P* = 0.064).

Patients in CCS 1 presented with Killip-class 1 were significantly more often (86%) compared with patients in CCS stages 2–4 (75%, 74%, and 60%, respectively; *P* < 0.001 for all). Killip-class was also significantly lower in patients in CCS 1 compared with those in CCS 2 (*P* = 0.012) and CCS 3 (*P* = 0.021). No significant difference in Killip-class was observed between CCS 2 and CCS 3 (*P* = 0.776). However, a significant difference in Killip-class was found between CCS 4 and CCS 1, CCS 2, or CCS 3 (all *P* < 0.001).

The right coronary artery (RCA) was more frequently the infarct-related artery in the CCS 1 (53%) and CCS 2 (47%) groups, while the left anterior descending (LAD) artery was most commonly the infarct-related artery among patients in CCS 3 (45%) and CCS 4 (50%) (*P* < 0.001).

Pre-interventional TIMI flow 0 also increased gradually from CCS stage 1 (30%) to CCS stages 2–4 (55%, 66%, and 77%, *P* < 0.001) with significant differences between groups (CCS 1 and CCS 2, *P* < 0.001; CCS 1 and CCS 3, *P* < 0.001; CCS 2 and CCS 3, *P* = 0.007; CCS 4 and CCS 1, *P* < 0.001; CCS 4 and CCS 2, *P* < 0.001; and CCS 4 and CCS 3, *P* = 0.002, respectively).

While an overall decrease in post-interventional TIMI flow 3 was observed from CCS stage 1 (96%) to CCS stages 2–4 (93%, 88%, and 89%, *P* = 0.005, *P* = 0.002 for trend), with significant differences between CCS 1 and CCS 3 (*P* = 0.002), CCS 1 and CCS 4 (*P* = 0.005), and CCS 2 and CCS 3 (*P* = 0.032), no significant differences were observed between CCS 1 and CCS 2, CCS 2 and CCS 4, and CCS 3 and CCS 4 (all *P* > 0.05).

**Figure 2:**
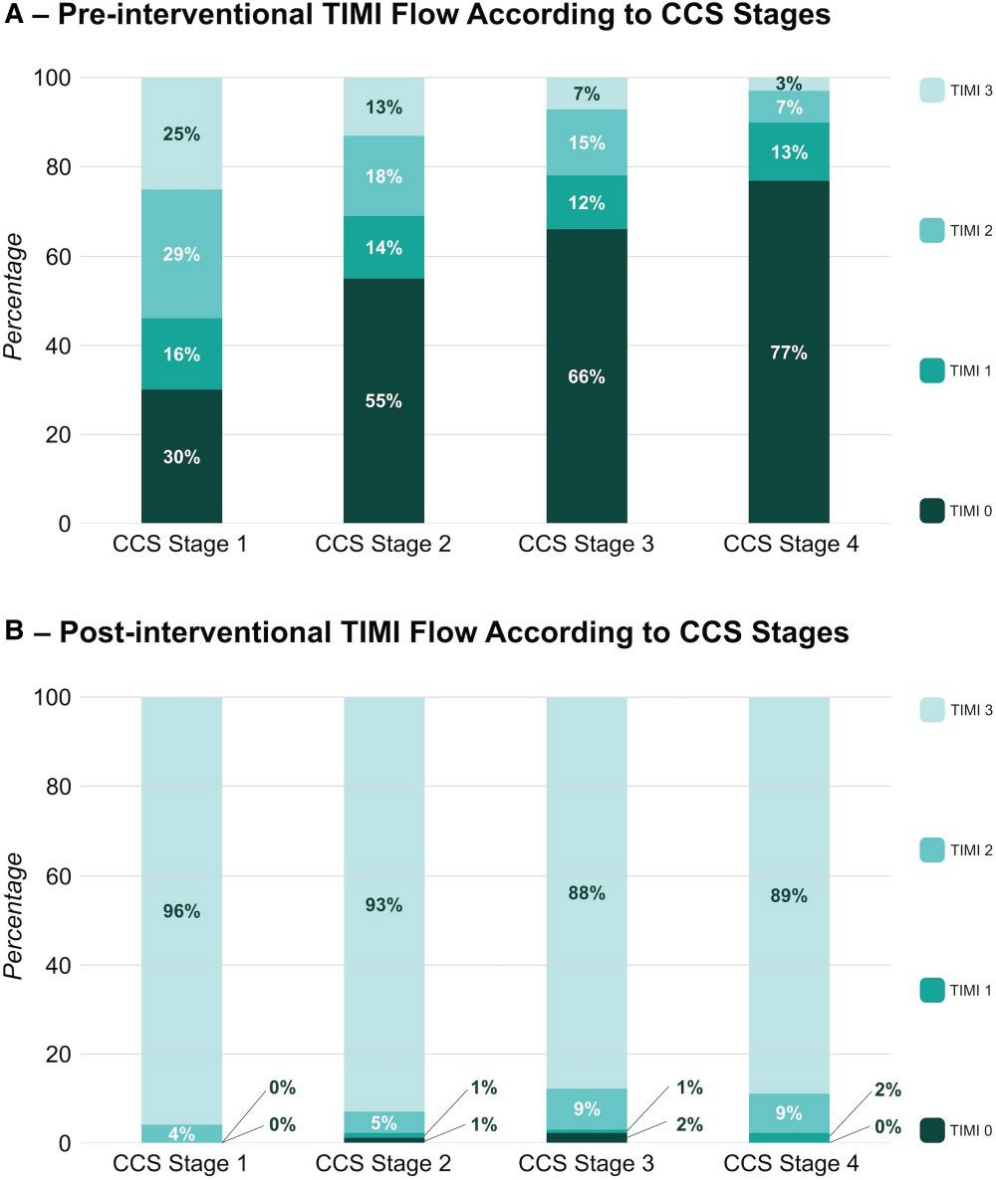
TIMI flow according to CCS stages. (A) Distribution of pre-interventional TIMI flow grades (0–3) across the four CCS stages in patients with STEMI. (B) Distribution of post-interventional TIMI flow grades following percutaneous coronary intervention (PCI) across the same CCS stages.

The association of pre- and post-interventional TIMI flow and CCS stages are provided in *Figures 2A* and *5B*, respectively.

### Biomarker release and AKI according to CCS stages

**Figure 3:**
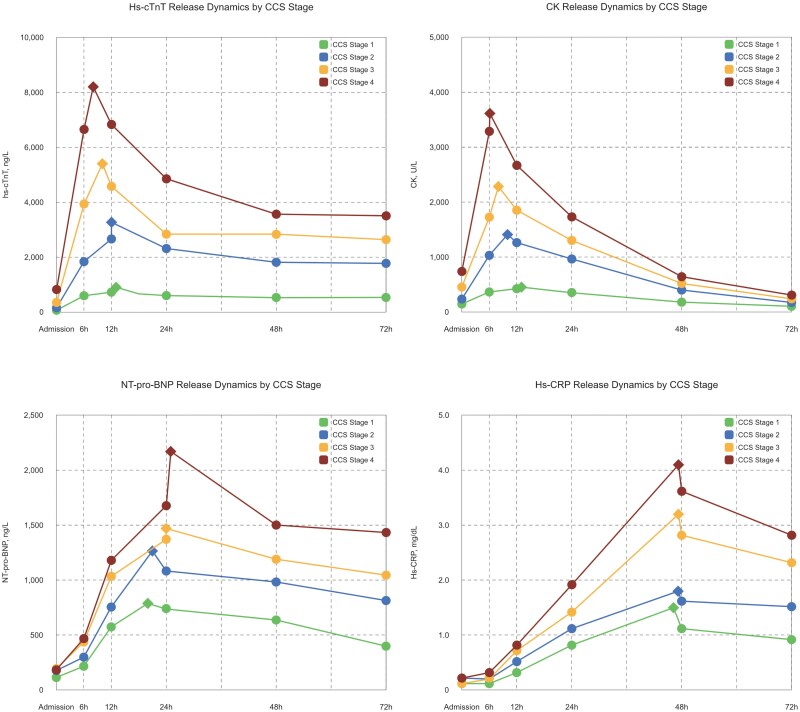
Biomarker release characteristics according to CCS stages. Four panels display the dynamic plasma concentration profiles of high-sensitivity cardiac troponin T (hs-cTnT), CK, NT-pro-BNP, and hs-CRP within the first 72 h after PCI in STEMI patients, stratified by CCS stage of myocardial tissue injury. A rhombus symbol (◇) indicates the peak value.

Peak biomarker values according to CCS stages are provided in *Table 1*. Detailed biomarker release patterns are provided in Supplementary data online, *Table S2* and *Figure 3*, respectively.

There was an increase in hs-cTnT and CK across all timeframes, in patients from CCS stages 1–4 (*Table 1*, *P* < 0.001). Furthermore, patients in CCS stage 4 peaked significantly earlier as compared with patients in CCS stage 1 [8 (6–12) and 13 (9–19) h, *P* < 0.001].

Similarly, a progressive increase in hs-CRP plasma concentrations and CCS stages was observed (*Table 1*). No difference in time-to-peak for hs-CRP between different CCS stages has been found (*P* = 0.726). NT-pro-BNP plasma concentrations were also significantly higher in patients with higher CCS stages. Time-to-peak NT-pro-BNP was significantly peaked later in patients with higher CCS stages (*P* = 0.020) (*Figure 3*).

No significant differences in creatinine levels were observed between CCS stages (all *P* > 0.05). Furthermore, CCS stages were not associated with the occurrence of AKI (*P* = 0.768).

### CMR imaging characteristics according to CCS stages

CMR scans were performed 3 days (IQR 2–5) after PCI, with no significant difference in the timing of CMR between CCS stages (*P* = 0.911). The median infarct size was 16% (IQR 8–26%) of LVMM. Infarct size varied significantly between CCS stages, with the smallest infarct size in CCS stage 1 [2% (IQR 0–3%) of LVMM] and a stepwise increase through CCS stage 2 [12% (IQR 8–18%)], CCS stage 3 [19% (IQR 14–26%)], and the largest in CCS stage 4 [27% (IQR 18–36%) of LVMM, *P* < 0.001]. Pairwise comparisons showed that infarct size was significantly different between each CCS stage (all *P* < 0.001).

Furthermore, LV ejection fraction (LVEF) decreased with advancing CCS stages (CCS 1: 57%, CCS 2: 52%, CCS 3: 50%, and CCS 4: 45%; *P* < 0.001), with significant differences observed between each CCS group (all *P* < 0.001). Similarly, the LV end-diastolic volume index (LVEDVi) increased with CCS stages (CCS 1: 74 mL/m², CCS 2: 78 mL/m², CCS 3: 80 mL/m², and CCS 4: 85 mL/m²; *P* < 0.001), as did the LV end-systolic volume index (LVESVi) (CCS 1: 31 mL/m², CCS 2: 37 mL/m², CCS 3: 41 mL/m², and CCS 4: 45 mL/m²; *P* < 0.001). Significant differences were found between each CCS group (all *P* < 0.001), except for LVEDVi between CCS 2 and CCS 3 (*P* = 0.118).

### Clinical outcome according to CCS stages

**Figure 4:**
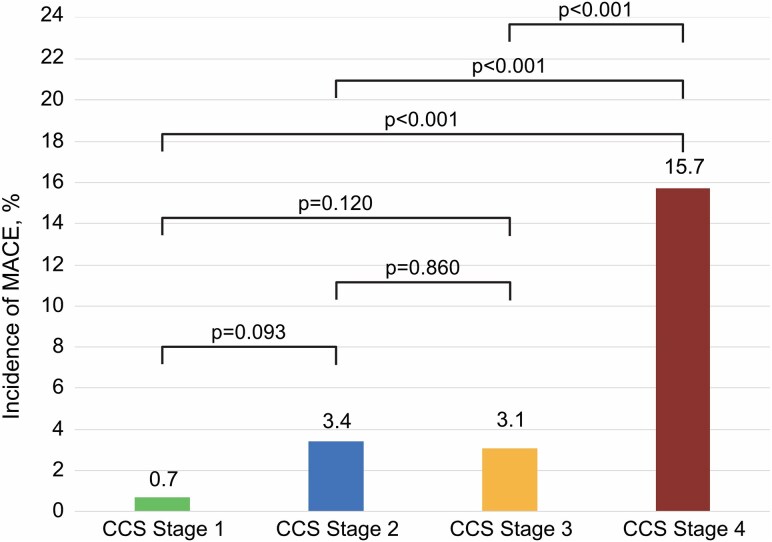
Incidence of MACE according to CCS stages. Bar chart showing the percentage of patients who experienced MACE defined as all-cause death or hospitalization for new congestive heart failure—within 12 months following STEMI, stratified by CCS myocardial tissue injury stages (1–4).

**Figure 5:**
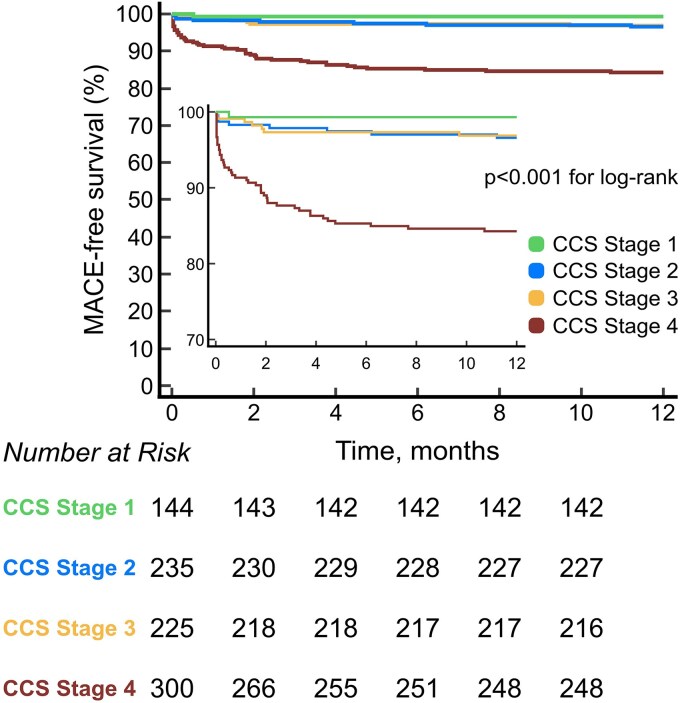
MACE-free survival according to CCS stages. Time-to-event analysis displaying 12-month MACE-free survival in patients with STEMI, stratified by CCS stage of myocardial tissue injury based on CMR imaging.

At 1 year, 63 (7.0%) patients experienced a MACE endpoint (14 all-cause deaths and 49 new congestive heart failure episodes). MACE was significantly more common in patients in CCS stage 4 (15.7%) as compared with patients in CCS stage 3 (3.1%), CCS stage 2 (3.4%), and CCS stage 1 (0.7%, all *P* < 0.001) (*Figure 4*). While there was a trend towards lower MACE in CCS 1—CCS 1 and 2 (*P* = 0.093) and CCS 1 and 3 (*P* = 0.120)—these differences did not reach statistical significance. Furthermore, CCS 2 and 3 had very similar numbers of MACE events [CCS 2 and 3 (*P* = 0.860)]. A Kaplan–Meier analysis plot of MACE-free survival according to CCS stages is shown in *Figure 5*.

In a sensitivity analysis excluding 31 patients with prior MI, results across CCS stages 1–4 remained unchanged: MACE rates were 0.7%, 2.7%, 2.7%, and 14.9% (*P* < 0.001); all-cause mortality was 0.7%, 0.9%, 0.9%, and 5.9% (*P* < 0.001).

All-cause death was observed in 26 (2.9%) patients. Death occurred significantly more often in patients in CCS stage 4 (6.3%) as compared with patients in CCS stage 3 (0.9%), CCS stage 2 (1.7%), and CCS stage 1 (0.7%, all *P* < 0.001). All-cause death was not significantly different between CCS stages 1–3 (CCS 1–3, group difference, *P* = 0.601; CCS 1 and 2, *P* = 0.404; CCS 1 and 3, *P* = 0.839; and CCS 2 and 3, *P* = 0.442) (*Graphical Abstract*).

In an exploratory analysis, CCS stage 3 was subdivided into CCS 3a [MVO below median (<1.17% of LVMM), *n* = 137] and CCS 3b [MVO ≥ median (≥1.17% of LVMM), *n* = 137]. MACE rates were 3.5% in CCS 3a and 2.7% in CCS 3b (*P* = 0.710).

As revealed by ROC analysis, CCS stages had a moderate to good predictive value for MACE [AUC 0.74 (95% CI: 0.68–0.80), *P* < 0.001] and mortality prediction [AUC 0.71 (95% CI: 0.61–0.80), *P* < 0.001] at 12 months. The combination of CCS stages and TIMI-risk score, as well as CCS stages and GRACE score 2.0 also provided an incremental prognostic value for the prediction of MACE [AUC 0.84 (95% CI: 0.80–0.89), *P* < 0.001 and AUC 0.88 (95% CI: 0.83–0.93), *P* < 0.001, respectively] in comparison to TIMI-risk score and GRACE score 2.0 alone [AUC difference, 0.04 (95% CI: 0.01–0.17, *P* = 0.027) and 0.05 (95% CI: 0.01–0.08, *P* = 0.005), respectively].

In addition, we compared the prognostic performance of CCS stages with the DERIVATE-ICM model^31^ and LGE mass alone. For MACE prediction, the DERIVATE-ICM model showed a comparable predictive value to CCS stages [AUC 0.76 (95% CI: 0.73–0.79); AUC difference 0.02, *P* = 0.623], while LGE mass alone had a lower predictive value [AUC 0.70 (95% CI: 0.67–0.73); AUC difference vs. CCS stages 0.05, *P* = 0.188 and vs. DERIVATE-ICM 0.06, *P* = 0.073]. For mortality prediction, CCS stages had a higher AUC than both the DERIVATE-ICM model [AUC 0.68 (95% CI: 0.65–0.71); AUC difference 0.03, *P* = 0.269] and LGE mass [AUC 0.64 (95% CI: 0.61–0.67); AUC difference 0.09, *P* = 0.109], with a smaller difference between DERIVATE-ICM and LGE mass (AUC difference 0.04, *P* = 0.509).

In Cox regression analysis, CCS stages remained an independent predictor of MACE at a median follow-up of 25 (±24 months) [HR: 2.18 (95% CI: 1.70–2.78), *P* < 0.001], after adjustment for patient characteristics, angiographic features, and global cardiac function parameters (*Table 2*). A complementary analysis including the TIMI and GRACE scores is provided in the Supplementary Data (Supplementary data online, *Table S5*).

**Table 2:** Cox regression analysis for the prediction of MACE

	Univariable		Multivariable	
	HR [95% CI]	*P*-value	HR [95% CI]	*P*-value
Age	1.88 [1.54–2.30]	**<0.001**	2.51 [1.69–3.73]	**<0**.**001**
Female sex	0.85 [0.71–1.01]	0.064	—	—
Body mass index	0.97 [0.79–1.17]	0.726	—	—
Hypertension	1.60 [1.30–1.95]	**<0**.**001**	1.73 [1.20–2.50]	**0**.**003**
Hyperlipidaemia	1.19 [0.98–1.45]	0.082	—	—
Diabetes mellitus	1.29 [1.11–1.50]	**<0**.**001**	—	—
Smoking	1.02 [0.84–1.25]	0.82	1.46 [1.03–2.07]	**0**.**032**
Admission Killip-class	1.90 [1.67–2.15]	**<0**.**001**	—	—
AKI	0.53 [0.12–2.26]	0.390	—	—
Total ischaemic time	1.06 [0.91–1.23]	0.445	—	—
Culprit lesion	1.09 [0.90–1.31]	0.395	—	—
Multivessel disease	1.48 [1.21–1.81]	**<0**.**001**	1.62 [1.15–2.29]	**0**.**006**
TIMI-flow 0 pre-PCI	1.46 [1.16–1.83]	**0**.**001**	1.69 [1.12–2.57]	**0**.**013**
TIMI-flow 3 post-PCI	0.84 [0.71–0.99]	**0**.**046**	—	—
LVEF	0.53 [0.44–0.64]	**<0**.**001**	—	—
LVEDVi	1.29 [1.06–1.57]	**0**.**013**	—	—
LVESVi	1.60 [1.35–1.89]	**<0**.**001**	—	—
CCS stages	2.18 [1.70–2.78]	**<0**.**001**	2.72 [1.74–4.42]	**<0**.**001**

Bold values indicate statistical significance defined as *P* < 0.05.

MACE, Major adverse cardiovascular event; HR, hazard ratio; CI, confidence interval; TIMI, thrombolysis in myocardial infarction; PCI, percutaneous coronary intervention; LVEF, left ventricular ejection fraction; LVEDVi, left ventricular end diastolic volume index; LVESVi, left ventricular end systolic volume index; CCS, Canadian Cardiovascular Society.

To address potential temporal heterogeneity due to evolving STEMI management, a stratified analysis comparing patients enrolled before (2009–17) and after (2018–22) the 2017 European Society of Cardiology (ESC) STEMI guideline update was performed.^32^ Prognostic patterns remained consistent across both cohorts: in the pre-guideline group, MACE occurred in 1.0%, 4.3%, 4.2%, and 16.6% across CCS stages 1–4 (*P* < 0.001), with mortality rates of 1.0%, 2.5%, 0.7%, and 6.6% (*P* = 0.007); in the post-guideline group, MACE rates were 0.0%, 1.4%, 1.2%, and 14.3% (*P* < 0.001), and mortality 0.0%, 0.0%, 1.2%, and 5.9% (*P* = 0.030).

## Discussion

Using data from three of the largest prospective cohort studies of patients with acute STEMI who underwent CMR at a standardized time point in the early post-MI phase, this study comprehensively analysed the prevalence, clinical characteristics, and prognostic implications of the recently proposed CCS classification in STEMI patients.

The key findings are as follows: (i) the prevalence of CCS stages was 16% for CCS stage 1, 27% for CCS stage 2, 25% for CCS stage 3, and 32% for CCS stage 4; (ii) clinical characteristics, as well as biomarker release patterns, differed between CCS stages; and (iii) the prognostic value of the CCS classification was significant and incremental to the TIMI-risk score in predicting clinical outcomes at 12 months. Patients with stage 1 had a very favourable prognosis (<1% MACE rate) and patients with stage 4 had by far the worst prognosis (>15% MACE rate). In contrast, patients with stages 2 and 3 had a very similar prognosis (3.4% and 3.1%) indicating limited discrimination.

Collectively, these data should be considered for future refinement of the classification and should also inform the use of CCS stages in future trial designs.

While the CCS classification encompasses the full spectrum of MI, the current study focuses on STEMI patients, as data on IMH in non-STEMI are lacking. Moreover, it provides the first detailed description of patient characteristics and clinical outcomes based on the proposed classification, utilizing individual data from three large prospective CMR studies. The observed distribution highlights not only the considerable variability in myocardial damage in STEMI patients but also the importance of patients with a haemorrhagic phenotype (CCS stage 4). This subgroup, comprising nearly one-third of all patients, significantly contributes to the overall population. While the distinctions between CCS 4, CCS 3, and CCS 2 can be clearly distinguished by different patterns of myocardial damage derived from multiparametric CMR, the classification of CCS 1 is confounded by the lack of established criteria for minimal myocardial damage.^17,20,33^ We have therefore evaluated different CCS 1 definitions (absence of LGE, LGE <1%, and LGE <5%) and observed consistent results. However, further studies are warranted to determine the best definition criteria for CCS 1.

In a subgroup of our study with available cardiac biomarker data, hs-cTnT levels increased with advancing CCS stages, which are consistent with the CCS expert consensus that associates higher troponin levels with progressive severity of myocardial tissue damage across CCS stages. In addition, the CCS expert consensus reports an earlier peak in troponin T concentrations in more advanced stages of CCS, a finding confirmed in our study.^17^ Our study is unique, as it extends cardiac biomarker analysis beyond troponin to include NT-pro-BNP, CK, and hs-CRP levels across CCS stages. NT-pro-BNP levels demonstrated a progressive increase with advancing CCS stages and peaked later in higher stages, likely reflecting ongoing myocardial dysfunction in the context of extensive tissue damage.^34^ This supports the concept that advanced stages are not only associated with greater initial injury but also with sustained functional impairment over time. Similarly, hs-CRP levels increased consistently with increasing CCS stages, highlighting differences in the inflammatory response.^35^

While patient characteristics such as TIMI-risk score, Killip-class, and biomarker profiles showed clear differences between CCS stages, post-interventional TIMI-flow and total ischaemic time did not consistently follow the expected trend of worsening with increasing CCS stage. These findings are particularly notable, as both parameters are established predictors of myocardial tissue damage,^36,37^ but may not fully reflect the progression of microvascular and structural injury captured by the CCS classification. This further emphasizes that successful revascularization of the epicardial coronary artery does not necessarily translate into successful microvascular blood flow.^38^ Additionally, the similarities in total ischaemic times suggest that microvascular injury is not solely determined by ischaemic injury to cardiomyocytes but rather by the fragile vascular endothelium and disrupted inter-endothelial junctions, which favour further cardiomyocyte damage after reperfusion therapy.^38,39^

In addition, female patients were less likely to present with a haemorrhagic MI (CCS 4). Although the reasons are not fully understood, it is known that men often experience STEMI at a younger age compared with women, who are typically post-menopausal at their first MI. Younger men are also more likely to be smokers, a factor linked to IMH after STEMI.^40^ These sex differences suggest underlying factors that warrant further investigation.

The clinical outcomes associated with each CCS stage demonstrate considerable variation in our analysis. Patients classified under CCS stage 1 typically exhibit a very favourable prognosis, also in our study, with a MACE rate of <1%. This again shows that if myocardial and microvascular injury is either absent or minimal, the likelihood of adverse cardiovascular outcomes is very low. The observation that these patients had a high rate of pre-PCI TIMI-flow >0 once again underscores the need for rapid and effective reperfusion.

Although CCS stages 2 and 3 can be clearly differentiated by the presence of MVO, these CCS groups had comparable clinical outcomes. This challenges the CCS expert consensus, which postulated a two- to four-fold increase in the risk of MACE between CCS stage 2 and stage 3.^17^ These findings are important and are very likely explained by the fact that previous studies investigating the prognostic implications of MVO were conducted based solely on the presence or absence of MVO, without considering a potential underlying co-occurrence of IMH. However, a clear distinction between MVO and IMH is essential to accurately assess their individual contributions to myocardial injury and outcome. In fact, in a recent study, we described three distinct microvascular injury patterns (no microvascular injury: MVO−/IMH−, presence of MVO: MVO+/IMH−, and presence of IMH: IMH+) and showed that prognosis does not differ significantly between patients with and without MVO, and that IMH is the main determinant of prognosis in patients with microvascular injury after reperfusion for STEMI.^7^ In contrast, Beek *et al*. demonstrated that the presence of MVO, but not IMH, independently predicted functional recovery at 4 months.^41^ Similarly, Demirkiran *et al*. assessed both markers and found no clear difference between MVO and IMH with respect to functional recovery or infarct size at 1 month.^42^ However, both studies were limited by small sample sizes and the use of T2-weighted imaging, which is susceptible to artefacts from oedema and less reliable for detecting IMH due to time-dependent changes in its signal characteristics.^43–46^ One possible explanation is the concept of microvascular stunning, where initial microvascular impairment does not lead to persistent adverse effects, implying that the impact of MVO alone may diminish over time without lasting consequences.^47^ Of note, previous studies have shown that MVO is a strong predictor of outcome when treated as a continuous variable.^48,49^ In accordance with this, the DERIVATE-ICM study recently demonstrated the strong prognostic relevance of continuous CMR parameters in patients with ischaemic cardiomyopathy.^31^ Yet the CCS classification only considers its presence or absence, neglecting the importance of quantification. This categorical approach does not distinguish between small and extensive MVO or IMH, potentially limiting its prognostic accuracy. In our cohort, CCS stages showed comparable prognostic performance to the DERIVATE-ICM model, which was evaluated in patients with established ischaemic cardiomyopathy. Future studies should, therefore, explore whether a refined CCS classification incorporating continuous CMR markers could further enhance risk stratification and clinical applicability.

In an exploratory analysis limited to CCS stage 3, patients were stratified by the median MVO extent into CCS 3a and CCS 3b. MACE rates were similar between these subgroups, indicating that a larger MVO extent without IMH does not confer a worse prognosis. While a three-tiered model distinguishing no or minimal infarction, infarction without IMH, and infarction with IMH may be conceptually appealing, this approach requires further validation. A first step, however, is the adoption of standardized imaging protocols. Given the variability introduced by differences in imaging timing^50^ and technique, such as the use of T2*-based CMR, which offers greater sensitivity for IMH detection than T2-weighted imaging,^43,44^ a uniform diagnostic approach will be essential for reliable and reproducible assessment.

An important finding of the present study concerns CCS stage 4, which represents a substantial proportion of our STEMI cohort (∼1/3). Patients in this stage exhibit by far the worst outcomes, with a MACE rate that is 15 times higher than those at CCS stage 1 and five times higher than those observed in CCS stages 2 or 3, respectively. The confirmation of the high incidence and poor prognosis associated with CCS stage 4 highlights a critical area for clinical intervention, where efforts to limit progression to this stage could significantly improve patient outcomes. Patients in CCS stage 4 represent a high-risk group that could benefit from intensified surveillance and early initiation of heart failure management. In addition, the haemorrhagic phenotype may serve as a valuable endpoint for future cardioprotection trials.

Future research should prioritize strategies that not only target early reperfusion but also focus on microvascular protection to prevent progression to CCS stage 4.

### Limitations

In the present study, STEMI patients suitable for CMR were enrolled, resulting in a non-sequential recruitment of participants. The exclusion of patients with unstable conditions at the time of image acquisition, such as high-risk STEMI patients limits the generalizability of our findings to all STEMI populations. Additionally, the broad enrolment period may introduce heterogeneity from evolving STEMI practices, potentially confounding outcome comparisons. The assessment of IMH was based on T2* mapping acquired in only three short-axis slices, which may not fully capture the extent of myocardial involvement. As a result, small regions of IMH may have been missed, and a comprehensive volumetric assessment of the entire left ventricle was not possible. Furthermore, patients with advanced renal dysfunction (CKD G4 or greater) were excluded from the study, which may limit the applicability of our findings to high-risk populations. Yet, only a small proportion of STEMI patients (∼2%) present with moderate to severe renal disease.^51^ In addition, because we focused on STEMI, it remains unknown whether these findings are applicable to the non-STEMI population. Biomarker profiles were limited to MARINA-STEMI, and functional follow-up data were not uniformly available. Additionally, cause-specific mortality was not consistently recorded, restricting insights into mechanisms of death. Finally, outcome analysis focused on a 1-year follow-up period. Although longer-term data were available in parts of the study population, they were not consistently collected across all cohorts and thus were not suitable for uniform analysis.

### Conclusion

This comprehensive study highlights the differences in clinical characteristics and provides valuable insights into the prognostic implications of the CCS stages. In particular, while stages 1 and 4 were associated with distinctly favourable and poor outcomes, respectively, stages 2 and 3 exhibited a comparable prognosis. This suggests that, although the CCS classification represents a valuable approach to stratify myocardial tissue injury, it may not fully differentiate intermediate stages such as CCS 2 and 3, where clinical and prognostic overlap is evident. These findings highlight the need for further refinement of the CCS classification and should be considered when using this classification in future studies.

## Supplementary Material

jeaf250_Supplementary_Data

## Data Availability

The data analysed in this study can be obtained from the corresponding authors with a reasonable request.

## References

[1] Christensen DM, Strange JE, El-Chouli M, Falkentoft AC, Malmborg M, Nouhravesh N et al Temporal trends in noncardiovascular morbidity and mortality following acute myocardial infarction. J Am Coll Cardiol 2023;82:971–81. 37648355 10.1016/j.jacc.2023.06.024

[2] Widimsky P, Wijns W, Fajadet J, de Belder M, Knot J, Aaberge L et al Reperfusion therapy for ST elevation acute myocardial infarction in Europe: description of the current situation in 30 countries. Eur Heart J 2010;31:943–57. 19933242 10.1093/eurheartj/ehp492 PMC2854523

[3] Luscher TF, Obeid S. From Eisenhower's heart attack to modern management: a true success story!. Eur Heart J 2017;38:3066–9. 29040468 10.1093/eurheartj/ehx569

[4] Carberry J, Marquis-Gravel G, 'Meara O, Docherty E, F K. Where are we with treatment and prevention of heart failure in patients post-myocardial infarction? JACC Heart Fail 2024;12:1157–65. 38878010 10.1016/j.jchf.2024.04.025

[5] Niccoli G, Burzotta F, Galiuto L, Crea F. Myocardial no-reflow in humans. J Am Coll Cardiol 2009;54:281–92. 19608025 10.1016/j.jacc.2009.03.054

[6] Lechner I, Reindl M, Tiller C, Holzknecht M, Troger F, Fink P et al Impact of COVID-19 pandemic restrictions on ST-elevation myocardial infarction: a cardiac magnetic resonance imaging study. Eur Heart J 2022;43:1141–53. 34632491 10.1093/eurheartj/ehab621 PMC8524546

[7] Lechner I, Reindl M, Stiermaier T, Tiller C, Holzknecht M, Oberhollenzer F et al Clinical outcomes associated with various microvascular injury patterns identified by CMR after STEMI. J Am Coll Cardiol 2024;83:2052–62. 38777509 10.1016/j.jacc.2024.03.408

[8] Pedersen F, Butrymovich V, Kelbaek H, Wachtell K, Helqvist S, Kastrup J et al Short- and long-term cause of death in patients treated with primary PCI for STEMI. J Am Coll Cardiol 2014;64:2101–8. 25457398 10.1016/j.jacc.2014.08.037

[9] Stone GW, Selker HP, Thiele H, Patel MR, Udelson JE, Ohman EM et al Relationship between infarct size and outcomes following primary PCI: patient-level analysis from 10 randomized trials. J Am Coll Cardiol 2016;67:1674–83. 27056772 10.1016/j.jacc.2016.01.069

[10] Reinstadler SJ, Stiermaier T, Reindl M, Feistritzer HJ, Fuernau G, Eitel C et al Intramyocardial haemorrhage and prognosis after ST-elevation myocardial infarction. Eur Heart J Cardiovasc Imaging 2019;20:138–46. 30165518 10.1093/ehjci/jey101

[11] Reinstadler SJ, Stiermaier T, Fuernau G, de Waha S, Desch S, Metzler B et al The challenges and impact of microvascular injury in ST-elevation myocardial infarction. Expert Rev Cardiovasc Ther 2016;14:431–43. 26794717 10.1586/14779072.2016.1135055

[12] Szummer K, Wallentin L, Lindhagen L, Alfredsson J, Erlinge D, Held C et al Improved outcomes in patients with ST-elevation myocardial infarction during the last 20 years are related to implementation of evidence-based treatments: experiences from the SWEDEHEART registry 1995–2014. Eur Heart J 2017;38:3056–65. 29020314 10.1093/eurheartj/ehx515 PMC5837507

[13] Liu T, Howarth AG, Chen Y, Nair AR, Yang H-J, Ren D et al Intramyocardial hemorrhage and the “Wave Front” of reperfusion injury compromising myocardial salvage. J Am Coll Cardiol 2022;79:35–48. 34991787 10.1016/j.jacc.2021.10.034 PMC13016961

[14] Lanza GA, Crea F. Primary coronary microvascular dysfunction: clinical presentation, pathophysiology, and management. Circulation 2010;121:2317–25. 20516386 10.1161/CIRCULATIONAHA.109.900191

[15] Camici PG, Crea F. Coronary microvascular dysfunction. N Engl J Med 2007;356:830–40. 17314342 10.1056/NEJMra061889

[16] Kumar A, Vora K, Bhatt DL, Dharmakumar R. The Canadian Cardiovascular Society Classification of acute atherothrombotic myocardial infarction provides a novel staging scheme based on tissue injury severity. Eur Heart J 2024;45:976–9. 38367011 10.1093/eurheartj/ehad821 PMC13017408

[17] Kumar A, Connelly K, Vora K, Bainey KR, Howarth A, Leipsic J et al The Canadian Cardiovascular Society classification of acute atherothrombotic myocardial infarction based on stages of tissue injury severity: an expert consensus statement. Can J Cardiol 2024;40:1–14. 37906238 10.1016/j.cjca.2023.09.020 PMC13016900

[18] Mayr A, Klug G, Reindl M, Lechner I, Tiller C, Holzknecht M et al Evolution of myocardial tissue injury: a CMR study over a decade after STEMI. JACC Cardiovasc Imaging 2022;15:1030–42. 35680211 10.1016/j.jcmg.2022.02.010

[19] Carrick D, Haig C, Ahmed N, McEntegart M, Petrie MC, Eteiba H et al Myocardial hemorrhage after acute reperfused ST-segment–elevation myocardial infarction. Circ Cardiovasc Imaging 2016;9:e004148. 26763281 10.1161/CIRCIMAGING.115.004148 PMC4718183

[20] Patel MR, Westerhout CM, Granger CB, Brener SJ, Fu Y, Siha H et al Aborted myocardial infarction after primary percutaneous coronary intervention: magnetic resonance imaging insights from the Assessment of Pexelizumab in Acute Myocardial Infarction (APEX-AMI) trial. Am Heart J 2013;165:226–33. 23351826 10.1016/j.ahj.2012.10.028

[21] Morrow DA, Antman EM, Charlesworth A, Cairns R, Murphy SA, de Lemos JA et al TIMI risk score for ST-elevation myocardial infarction: a convenient, bedside, clinical score for risk assessment at presentation: an intravenous nPA for treatment of infarcting myocardium early II trial substudy. Circulation 2000;102:2031–7. 11044416 10.1161/01.cir.102.17.2031

[22] Fox KA, Fitzgerald G, Puymirat E, Huang W, Carruthers K, Simon T et al Should patients with acute coronary disease be stratified for management according to their risk? Derivation, external validation and outcomes using the updated GRACE risk score. BMJ Open 2014;4:e004425. 10.1136/bmjopen-2013-004425 PMC3931985 24561498

[23] Reindl M, Tiller C, Holzknecht M, Lechner I, Beck A, Plappert D et al Prognostic implications of global longitudinal strain by feature-tracking cardiac magnetic resonance in ST-elevation myocardial infarction. Circ Cardiovasc Imaging 2019;12:e009404. 31679391 10.1161/CIRCIMAGING.119.009404

[24] Carrick D, Haig C, Rauhalammi S, Ahmed N, Mordi I, McEntegart M et al Pathophysiology of LV remodeling in survivors of STEMI: inflammation, remote myocardium, and prognosis. JACC Cardiovasc Imaging 2015;8:779–89. 26093923 10.1016/j.jcmg.2015.03.007 PMC4509710

[25] Lechner I, Reindl M, Oberhollenzer F, Tiller C, Holzknecht M, Fink P et al Association of dysglycaemia with persistent infarct core iron in patients with acute ST-segment elevation myocardial infarction. J Cardiovasc Magn Reson 2024;26:100996. 38237898 10.1016/j.jocmr.2024.100996 PMC11211234

[26] Reinstadler SJ, Kronbichler A, Reindl M, Feistritzer HJ, Innerhofer V, Mayr A et al Acute kidney injury is associated with microvascular myocardial damage following myocardial infarction. Kidney Int 2017;92:743–50. 28412022 10.1016/j.kint.2017.02.016

[27] Khwaja A . KDIGO clinical practice guidelines for acute kidney injury. Nephron Clin Pract 2012;120:c179–84. 22890468 10.1159/000339789

[28] Lechner I, Reindl M, Tiller C, Holzknecht M, Fink P, Plangger J et al Association between inflammation and left ventricular thrombus formation following ST-elevation myocardial infarction. Int J Cardiol 2022;361:1–6. 35533756 10.1016/j.ijcard.2022.05.009

[29] DeLong ER, DeLong DM, Clarke-Pearson DL. Comparing the areas under two or more correlated receiver operating characteristic curves: a nonparametric approach. Biometrics 1988;44:837–45. 3203132

[30] Rice ME, Harris GT. Comparing effect sizes in follow-up studies: ROC area, Cohen’s D, and R. Law Hum Behav 2005;29:615–20. 16254746 10.1007/s10979-005-6832-7

[31] Pontone G, Guaricci AI, Fusini L, Baggiano A, Guglielmo M, Muscogiuri G et al Cardiac magnetic resonance for prophylactic implantable-cardioverter defibrillator therapy in ischemic cardiomyopathy: the DERIVATE-ICM International Registry. JACC Cardiovasc Imaging 2023;16:1387–400. 37227329 10.1016/j.jcmg.2023.03.015

[32] Ibanez B, James S, Agewall S, Antunes MJ, Bucciarelli-Ducci C, Bueno H et al 2017 ESC Guidelines for the management of acute myocardial infarction in patients presenting with ST-segment elevation: The Task Force for the management of acute myocardial infarction in patients presenting with ST-segment elevation of the European Society of Cardiology (ESC). Eur Heart J 2018;39:119–77. 28886621 10.1093/eurheartj/ehx393

[33] Eitel I, Desch S, Sareban M, Fuernau G, Gutberlet M, Schuler G et al Prognostic significance and magnetic resonance imaging findings in aborted myocardial infarction after primary angioplasty. Am Heart J 2009;158:806–13. 19853702 10.1016/j.ahj.2009.08.025

[34] Mayr A, Mair J, Schocke M, Klug G, Pedarnig K, Haubner BJ et al Predictive value of NT-pro BNP after acute myocardial infarction: relation with acute and chronic infarct size and myocardial function. Int J Cardiol 2011;147:118–23. 19896736 10.1016/j.ijcard.2009.09.537

[35] Reindl M, Reinstadler SJ, Feistritzer HJ, Klug G, Tiller C, Mair J et al Relation of inflammatory markers with myocardial and microvascular injury in patients with reperfused ST-elevation myocardial infarction. Eur Heart J Acute Cardiovasc Care 2017;6:640–9. 27440935 10.1177/2048872616661691

[36] Greulich S, Mayr A, Gloekler S, Seitz A, Birkmeier S, Schaufele T et al Time-dependent myocardial necrosis in patients with ST-segment-elevation myocardial infarction without angiographic collateral flow visualized by cardiac magnetic resonance imaging: results from the Multicenter STEMI-SCAR Project. J Am Heart Assoc 2019;8:e012429. 31181983 10.1161/JAHA.119.012429 PMC6645633

[37] Joost A, Stiermaier T, Eitel C, Fuernau G, Waha SD, Desch S et al Impact of initial culprit vessel flow on infarct size, microvascular obstruction, and myocardial salvage in acute reperfused ST-elevation myocardial infarction. Am J Cardiol 2016;118:1316–22. 27600465 10.1016/j.amjcard.2016.07.056

[38] Vora KP, Kumar A, Krishnam MS, Prato FS, Raman SV, Dharmakumar R. Microvascular obstruction and intramyocardial hemorrhage in reperfused myocardial infarctions: pathophysiology and clinical insights from imaging. JACC Cardiovasc Imaging 2024;17:795–810. 38613553 10.1016/j.jcmg.2024.02.003 PMC13159459

[39] Chistiakov DA, Orekhov AN, Bobryshev YV. Endothelial barrier and its abnormalities in cardiovascular disease. Front Physiol 2015;6:365. 26696899 10.3389/fphys.2015.00365 PMC4673665

[40] Haig C, Carrick D, Carberry J, Mangion K, Maznyczka A, Wetherall K et al Current smoking and prognosis after acute ST-segment elevation myocardial infarction: new pathophysiological insights. JACC Cardiovasc Imaging 2019;12:993–1003. 30031700 10.1016/j.jcmg.2018.05.022 PMC6547246

[41] Beek AM, Nijveldt R, van Rossum AC. Intramyocardial hemorrhage and microvascular obstruction after primary percutaneous coronary intervention. Int J Cardiovasc Imaging 2010;26:49–55. 19757151 10.1007/s10554-009-9499-1 PMC2795157

[42] Demirkiran A, Robbers L, van der Hoeven NW, Everaars H, Hopman L, Janssens GN et al The dynamic relationship between invasive microvascular function and microvascular injury indicators, and their association with left ventricular function and infarct size at 1-month after reperfused ST-segment-elevation myocardial infarction. Circ Cardiovasc Interv 2022;15:892–902. 36305318 10.1161/CIRCINTERVENTIONS.122.012081

[43] Zia MI, Ghugre NR, Connelly KA, Strauss BH, Sparkes JD, Dick AJ et al Characterizing myocardial edema and hemorrhage using quantitative T2 and T2* mapping at multiple time intervals post ST-segment elevation myocardial infarction. Circ Cardiovasc Imaging 2012;5:566–72. 22744938 10.1161/CIRCIMAGING.112.973222

[44] Kali A, Tang RL, Kumar A, Min JK, Dharmakumar R. Detection of acute reperfusion myocardial hemorrhage with cardiac MR imaging: T2 versus T2. Radiology 2013;269:387–95. 23847253 10.1148/radiol.13122397 PMC3807083

[45] Ibanez B, Aletras AH, Arai AE, Arheden H, Bax J, Berry C et al Cardiac MRI endpoints in myocardial infarction experimental and clinical trials. JACC Scientific Expert Panel. J Am Coll Cardiol 2019;74:238–56. 10.1016/j.jacc.2019.05.024 PMC7363031 31296297

[46] Hamirani YS, Wong A, Kramer CM, Salerno M. Effect of microvascular obstruction and intramyocardial hemorrhage by CMR on LV remodeling and outcomes after myocardial infarction: a systematic review and meta-analysis. JACC Cardiovasc Imaging 2014;7:940–52. 25212800 10.1016/j.jcmg.2014.06.012 PMC4301583

[47] Dharmakumar R, Kumar A. Hemorrhagic myocardial infarction: light after 50 years in the tunnel. J Am Coll Cardiol 2024;83:2063–5. 38777510 10.1016/j.jacc.2024.03.413 PMC12983092

[48] de Waha S, Patel MR, Granger CB, Ohman EM, Maehara A, Eitel I et al Relationship between microvascular obstruction and adverse events following primary percutaneous coronary intervention for ST-segment elevation myocardial infarction: an individual patient data pooled analysis from seven randomized trials. Eur Heart J 2017;38:3502–10. 29020248 10.1093/eurheartj/ehx414

[49] Husser O, Monmeneu JV, Sanchis J, Nunez J, Lopez-Lereu MP, Bonanad C et al Cardiovascular magnetic resonance-derived intramyocardial hemorrhage after STEMI: Influence on long-term prognosis, adverse left ventricular remodeling and relationship with microvascular obstruction. Int J Cardiol 2013;167:2047–54. 22682700 10.1016/j.ijcard.2012.05.055

[50] Carrick D, Haig C, Ahmed N, Rauhalammi S, Clerfond G, Carberry J et al Temporal evolution of myocardial hemorrhage and edema in patients after acute ST-segment elevation myocardial infarction: pathophysiological insights and clinical implications. J Am Heart Assoc 2016;5:e002834. 26908408 10.1161/JAHA.115.002834 PMC4802451

[51] Thrane PG, Olesen KKW, Thim T, Gyldenkerne C, Hansen MK, Stødkilde-Jørgensen N et al 10-year mortality after ST-segment elevation myocardial infarction compared to the general population. J Am Coll Cardiol 2024;83:2615–25. 38897670 10.1016/j.jacc.2024.04.025

