# Supplementary material for: Associations and prognostic implications of myocardial tissue injury stages in ST-elevation myocardial infarction using the Canadian Cardiovascular Society classification
Source: Eur Heart J Cardiovasc Imaging. 2025 Aug 22;27(4):638–49. doi: 10.1093/ehjci/jeaf250 (PMC13021278; doi:10.1093/ehjci/jeaf250)
Supplement: jeaf250_Supplementary_Data [file jeaf250_supplementary_data.docx]

# Supplementary Data

# Table S1: DETAILED INCLUSION AND EXCLUSION CRITERIA OF THE INCLUDED STUDIES

| Study | Inclusion Criteria | Exclusion Criteria |
| --- | --- | --- |
| MARINA-STEMI  (NCT04113356) | - Patients ≥18 years of age - First-ever STEMI defined as having symptoms of ischemia and ST-segment elevation of at least 0.1 mV in two contiguous extremity leads or at least 0.2 mV in two contiguous precordial leads - Treated by primary PCI within 12 hours after symptom onset | - Contraindications for CMR (estimated glomerular filtration rate <30 mL/min/1.73 m^2^, Killip-classification higher than II at the time of cardiac MRI, pacemaker, claustrophobia, orbital foreign body, cerebral aneurysm clip, and known or suggested contrast agent allergy to gadolinium) - Patients with a history of previous myocardial infarction |
| BHF MR-MI  (NCT02072850) | - Acute STEMI - Treated by emergency PCI | - Major systemic illness (e.g. cancer limiting survival <6 months) - Metallic implant (e.g. cochlear implant) - Metallic foreign body - Pregnancy |
| HEM-CMR  (Not registered) | - Patients ≥18 years of age - Consecutive STEMI patients - Treated by primary PCI within 12 hours after symptom onset | - Estimated glomerular filtration rate <30 mL/min/1.73 m^2^ - Contraindications to CMR - Hemodynamic instability not allowing CMR acquisition. |

Abbreviations: MARINA=Magnetic Resonance Imaging In Acute ST-Elevation Myocardial Infarction, STEMI=ST-elevation myocardial infarction, PCI: Percutaneous coronary intervention, CMR=Cardiac magnetic resonance, MRI=Magnetic resonance imaging, BHF=British Heart Foundation, MR=Magnetic resonance, MI=Myocardial infarction, HEM-CMR= HEM-CMR=Haemorrhage Assessed by Cardiac Magnetic Resonance in ST-Elevation Myocardial Infarction.

**TABLE S2: BIOMARKER RELEASE PATTERNS ACCORDING TO CCS STAGES – CCS STAGE 1 DEFINED BY LGE <5% of LVMM**

|  | Total population  (n=1,109) | CCS 1  (n=179, 16%) | CCS 2  (n=297, 27%) | CCS 3  (n=274, 25%) | CCS 4  (n=359, 32%) | p-value |
| --- | --- | --- | --- | --- | --- | --- |
| Hs-cTnT, admission, ng/L | 214 [45-1115] | 70 [28-207] | 160 [31-429] | 350 [63-1601] | 823 [78-2816] | **<0.001** |
| Hs-cTnT, 6h, ng/L | 3155 [1124-6628] | 596 [224-1302] | 1833 [988-3282] | 3930 [1735-6884] | 6651 [4368-12038] | **<0.001** |
| Hs-cTnT, 12h, ng/L | 3936 [1764-6765] | 725 [300-1656] | 2658 [1574-4481] | 4576 [3044-6326] | 6830 [4377-9637] | **<0.001** |
| Hs-cTnT, 24h, ng/L | 3077 [1475-5177] | 602 [342-1236] | 2309 [1305-3849] | 3628 [2275-5087] | 4853 [3154-7106] | **<0.001** |
| Hs-cTnT, 48h, ng/L | 2317 [1260-3994] | 525 [288-893] | 1809 [1119-3070] | 2832 [1838-3963] | 3559 [2313-5561] | **<0.001** |
| Hs-cTnT, 72h, ng/L | 2270 [1281-3896] | 534 [308-1038] | 1770 [1127-2655] | 2639 [1771-3786] | 3503 [2313-5335] | **<0.001** |
| Hs-cTnT, time-to-peak, hours | 11 [7-15] | 13 [9-18] | 12 [9-17] | 10 [6-16] | 8 [6-12] | **<0.001** |
| CK, admission, U/L | 305 [136-921] | 147 [96-281] | 236 [133-441] | 455 [158-1085] | 739 [215-1649] | **<0.001** |
| CK, 6h, U/L | 1576 [742-3177] | 366 [182-669] | 1032 [673-1735] | 1727 [1002-3088] | 3288 [1843-4518] | **<0.001** |
| CK, 12h, U/L | 1618 [916-2786] | 425 [240-788] | 1264 [809-1942] | 1853 [1253-2853] | 2669 [1667-3865] | **<0.001** |
| CK, 24h, U/L | 1133 [608-1857] | 356 [193-625] | 966 [586-1422] | 1303 [861-1910] | 1732 [1072-2620] | **<0.001** |
| CK, 48h, U/L | 465 [267-769] | 181 [101-317] | 401 [226-626] | 520 [322-746] | 643 [395-1021] | **<0.001** |
| CK, 72h, U/L | 222 [136-371] | 105 [76-175] | 175 [129-338] | 241 [169-375] | 309 [209-498] | **<0.001** |
| CK, time-to-peak, hours | 8 [6-13] | 13 [8-17] | 10 [7-15] | 8 [6-13] | 6 [5-9] | **<0.001** |
| NT-pro-BNP, admission, ng/L | 170 [64-570] | 114 [50-283] | 177 [59-458] | 194 [66-804] | 183 [74-821] | **0.006** |
| NT-pro-BNP, 6h, ng/L | 355 [155-1007] | 216 [92-582] | 299 [130-836] | 437 [162-1046] | 468 [208-1446] | **<0.001** |
| NT-pro-BNP, 12h, ng/L | 928 [498-1815] | 574 [251-1189] | 755 [463-1590] | 1034 [564-2013] | 1180 [701-2216] | **<0.001** |
| NT-pro-BNP, 24h, ng/L | 1302 [709-2332] | 741 [375-1509] | 1088 [645-1954] | 1371 [750-2626] | 1677 [1015-2932] | **<0.001** |
| NT-pro-BNP, 48h, ng/L | 1132 [607-2095] | 637 [293-1035] | 983 [500-1719] | 1190 [656-2404] | 1501 [920-2862] | **<0.001** |
| NT-pro-BNP, 72h, ng/L | 1009 [469-1797] | 399 [185-698] | 814 [427-1494] | 1045 [513-2035] | 1433 [803-2627] | **<0.001** |
| NT-pro-BNP, time-to-peak, hours | 23 [17-44] | 20 [15-36] | 21 [17-39] | 24 [16-44] | 25 [19-45] | **0.020** |
| Creatinine, admission, U/L | 1.0 [0.8-1.1] | 1.0 [0.9-1.1] | 0.9 [0.8-1.1] | 1.0 [0.8-1.1] | 0.9 [0.8-1.1] | 0.854 |
| Creatinine, 6h, mg/dL | 0.9 [0.8-1.0] | 0.9 [0.8-1.0] | 0.9 [0.8-1.0] | 0.9 [0.8-1.0] | 0.9 [0.8-1.0] | 0.779 |
| Creatinine, 12h, mg/dL | 0.9 [0.8-1.0] | 0.9 [0.9-1.0] | 0.9 [0.8-1.0] | 0.9 [0.8-1.0] | 0.9 [0.8-1.1] | 0.648 |
| Creatinine, 24h, mg/dL | 0.9 [0.8-1.1] | 0.9 [0.8-1.0] | 0.9 [0.8-1.1] | 1.0 [0.8-1.1] | 0.9 [0.8-1.1] | 0.962 |
| Creatinine, 48h, mg/dL | 1.0 [0.9-1.1] | 1.0 [0.9-1.1] | 1.0 [0.9-1.1] | 1.0 [0.9-1.1] | 1.0 [0.9-1.1] | 0.979 |
| Creatinine, 72h, mg/dL | 1.0 [0.9-1.1] | 1.0 [0.9-1.1] | 1.0 [0.8-1.1] | 1.0 [0.9-1.1] | 1.0 [0.9-1.1] | 0.883 |
| Creatinine, time-to-peak, hours | 49 [17-78] | 40 [6-65] | 48 [17-78] | 50 [20-78] | 53 [19-78] | **0.047** |
| Hs-CRP, admission, ng/dL | 0.3 [0.1-0.5] | 0.2 [0.1-0.4] | 0.3 [0.1-0.6] | 0.2 [0.1-0.5] | 0.3 [0.1-0.6] | **0.029** |
| Hs-CRP, 6h, mg/dL | 0.3 [0.2-0.7] | 0.2 [0.1-0.6] | 0.3 [0.2-0.6] | 0.3 [0.2-0.6] | 0.4 [0.2-0.8] | **0.005** |
| Hs-CRP, 12h, mg/dL | 0.7 [0.4-1.3] | 0.4 [0.2-1.0] | 0.6 [0.3-1.0] | 0.8 [0.4-1.4] | 0.9 [0.5-1.6] | **<0.001** |
| Hs-CRP, 24h, mg/dL | 1.4 [0.7-2.7] | 0.9 [0.4-1.6] | 1.2 [0.7-1.8] | 1.5 [0.8-3.2] | 2.0 [1.1-3.6] | **<0.001** |
| Hs-CRP, 48h, mg/dL | 2.5 [1.2-4.4] | 1.2 [0.8-2.4] | 1.7 [1.0-3.2] | 2.9 [1.4-4.8] | 3.7 [2.2-6.8] | **<0.001** |
| Hs-CRP, 72h, mg/dL | 2.1 [1.1-3.9] | 1.0 [0.5-2.4] | 1.6 [0.8-3.0] | 2.4 [1.3-4.3] | 2.9 [1.7-5.8] | **<0.001** |
| Hs-CRP, time-to-peak, hours | 47 [37-59] | 45 [34-59] | 46 [36-59] | 47 [40-60] | 47 [37-57] | 0.726 |

Abbreviations: CCS=Canadian Cardiovascular Society, Hs-cTnT=High-sensitivity cardiac troponin T, CK=Creatine kinase, NT-pro-BNP=N-terminal pro-B-type natriuretic peptide, Hs-CRP=High-sensitive C-reactive protein, *data available for patients in the MARINA-STEMI study (n=600).

# Table S3: BASELINE CHARACTERISTICS ACCORDING TO CCS STAGES – CCS STAGE 1 DEFINED BY ABSENCE OF LGE

|  | Total population  (n=1,109) | CCS 1  (n=49, 4%) | CCS 2  (n=427, 39%) | CCS 3  (n=274, 25%) | CCS 4  (n=359, 32%) | p-value |
| --- | --- | --- | --- | --- | --- | --- |
| Patient characteristics |  |  |  |  |  |  |
| Age, years | 58 [51-68] | 60 [51-71] | 59 [52-68] | 58 [51-68] | 58 [52-68] | 0.872 |
| Female sex, n (%) | 242 (22) | 11 (22) | 100 (23) | 70 (26) | 61 (17) | 0.051 |
| Body mass index, kg/m^2^ | 27 [25-29] | 26 [25-30] | 27 [25-30] | 26 [25-29] | 27 [25-29] | 0.548 |
| Hypertension, n (%) | 503 (45) | 27 (55) | 180 (42) | 138 (50) | 158 (44) | 0.083 |
| Hyperlipidaemia, n (%) | 476 (43) | 27 (55) | 196 (46) | 108 (40) | 145 (40) | 0.082 |
| Diabetes mellitus, n (%) | 117 (11) | 9 (18) | 41 (10) | 26 (10) | 41 (11) | 0.244 |
| Smoker, n (%) | 608 (55) | 27 (55) | 238 (56) | 138 (51) | 205 (58) | 0.402 |
| TIMI-risk score | 3 [2-5] | 3 [1-5] | 3 [1-4] | 3 [2-5] | 3 [2-5] | **0.001** |
| GRACE 2.0 score^†^ | 108 [93-125] | 111 [92-134] | 104 [90-121] | 107 [93-123] | 111 [94-129] | 0.030 |
| Admission Killip-class |  |  |  |  |  | **<0.001** |
| 1 | 793 (72) | 40 (82) | 336 (79) | 203 (74) | 214 (60) |  |
| 2 | 281 (25) | 8 (16) | 87 (20) | 67 (25) | 119 (33) |  |
| 3 | 26 (2) | 0 (0) | 4 (1) | 3 (1) | 19 (5) |  |
| 4 | 9 (1) | 1 (2) | 0 (0) | 1 (<1) | 7 (2) |  |
| Acute kidney injury, n (%)* | 27 (2.4) | 1 (2.0) | 9 (2.1) | 8 (2.9) | 9 (2.5) | 0.918 |
| Total ischemic time, min | 179 [114-311] | 135 [95-259] | 171 [172-287] | 182 [115-310] | 200 [121-337] | **0.006** |
|  |  |  |  |  |  |  |
| Biomarkers characteristics |  |  |  |  |  |  |
| Hs-cTnT, admission, ng/L | 214 [45-1115] | 73 [29-379] | 126 [31-324] | 350 [63-1601] | 823 [78-2816] | **<0.001** |
| Hs-cTnT, 6h, ng/L | 3155 [1124-6628] | 356 [124-1413] | 1363 [596-2749] | 3930 [1735-6884] | 6651 [4368-12038] | **<0.001** |
| Hs-cTnT, 12h, ng/L | 3936 [1764-6765] | 418 [168-1609] | 1978 [984-3557] | 4576 [3044-6326] | 6830 [4377-9637] | **<0.001** |
| Hs-cTnT, 24h, ng/L | 3077 [1475-5177] | 312 [166-1126] | 1517 [821-3138] | 3628 [2275-5087] | 4853 [3154-7106] | **<0.001** |
| Hs-cTnT, 48h, ng/L | 2317 [1260-3994] | 261 [135-1051] | 1371 [742-2309] | 2832 [1838-3963] | 3559 [2313-5561] | **<0.001** |
| Hs-cTnT, 72h, ng/L | 2270 [1281-3896] | 308 [142-704] | 1392 [753-2230] | 2639 [1771-3786] | 3503 [2313-5335] | **<0.001** |
| Hs-cTnT, peak, ng/L | 4776 [2155-8076] | 660 [248-1808] | 2218 [1221-4541] | 5402 [3466-7533] | 8198 [5199-13816] | **<0.001** |
| Hs-cTnT, time-to-peak, hours | 11 [7-15] | 8 [6-39] | 13 [9-17] | 10 [6-16] | 8 [6-12] | **<0.001** |
| CK, admission, U/L | 305 [136-921] | 140 [79-390] | 198 [120-411] | 455 [158-1085] | 739 [215-1649] | **<0.001** |
| CK, 6h, U/L | 1576 [742-3177] | 225 [99-686] | 841 [431-1524] | 1727 [1002-3088] | 3288 [1843-4518] | **<0.001** |
| CK, 12h, U/L | 1618 [916-2786] | 249 [124-793] | 984 [544-1586] | 1853 [1253-2853] | 2669 [1667-3865] | **<0.001** |
| CK, 24h, U/L | 1133 [608-1857] | 214 [120-784] | 738 [403-1235] | 1303 [861-1910] | 1732 [1072-2620] | **<0.001** |
| CK, 48h, U/L | 465 [267-769] | 106 [50-421] | 312 [176-555] | 520 [322-746] | 643 [395-1021] | **<0.001** |
| CK, 72h, U/L | 222 [136-371] | 97 [57-174] | 158 [107-281] | 241 [169-375] | 309 [209-498] | **<0.001** |
| CK, peak, U/L | 1913 [980-3508] | 293 [158-841] | 1093 [581-1805] | 2284 [1482-3345] | 3614 [2237-4736] | **<0.001** |
| CK, time-to-peak, hours | 8 [6-13] | 10 [8-17] | 11 [8-15] | 8 [6-13] | 6 [5-9] | **<0.001** |
| NT-pro-BNP, admission, ng/L | 170 [64-570] | 149 [55-928] | 154 [50-365] | 194 [66-804] | 183 [74-821] | **0.020** |
| NT-pro-BNP, 6h, ng/L | 355 [155-1007] | 257 [151-1395] | 279 [111-621] | 437 [162-1046] | 468 [208-1446] | **<0.001** |
| NT-pro-BNP, 12h, ng/L | 928 [498-1815] | 730 [301-2680] | 721 [383-1353] | 1034 [564-2013] | 1180 [701-2216] | **<0.001** |
| NT-pro-BNP, 24h, ng/L | 1302 [709-2332] | 692 [307-2370] | 964 [565-1734] | 1371 [750-2626] | 1677 [1015-2932] | **<0.001** |
| NT-pro-BNP, 48h, ng/L | 1132 [607-2095] | 667 [293-1427] | 852 [450-1528] | 1190 [656-2404] | 1501 [920-2862] | **<0.001** |
| NT-pro-BNP, 72h, ng/L | 1009 [469-1797] | 617 [281-1161] | 642 [324-1342] | 1045 [513-2035] | 1433 [803-2627] | **<0.001** |
| NT-pro-BNP, peak, ng/L | 1453 [758-2716] | 941 [349-2864] | 1095 [615-1805] | 1470 [838-3219] | 2107 [1179-3651] | **<0.001** |
| NT-pro-BNP, time-to-peak, hours | 23 [17-44] | 20 [14-39] | 21 [17-37] | 24 [16-44] | 25 [19-45] | **0.040** |
| Creatinine, admission, U/L | 1.0 [0.8-1.1] | 0.9 [0.8-1.0] | 1.0 [0.8-1.1] | 1.0 [0.8-1.1] | 0.9 [0.8-1.1] | 0.906 |
| Creatinine, 6h, mg/dL | 0.9 [0.8-1.0] | 0.9 [0.8-1.0] | 0.9 [0.8-1.0] | 0.9 [0.8-1.0] | 0.9 [0.8-1.0] | 0.952 |
| Creatinine, 12h, mg/dL | 0.9 [0.8-1.0] | 1.0 [0.9-1.0] | 0.9 [0.8-1.0] | 0.9 [0.8-1.0] | 0.9 [0.8-1.1] | 0.806 |
| Creatinine, 24h, mg/dL | 0.9 [0.8-1.1] | 1.0 [0.9-1.0] | 0.9 [0.8-1.1] | 1.0 [0.8-1.1] | 0.9 [0.8-1.1] | 0.806 |
| Creatinine, 48h, mg/dL | 1.0 [0.9-1.1] | 1.0 [0.9-1.1] | 1.0 [0.9-1.1] | 1.0 [0.9-1.1] | 1.0 [0.9-1.1] | 0.936 |
| Creatinine, 72h, mg/dL | 1.0 [0.9-1.1] | 1.0 [1.0-1.1] | 1.0 [0.8-1.1] | 1.0 [0.9-1.1] | 1.0 [0.9-1.1] | 0.977 |
| Creatinine, peak, mg/dL | 1.1 [1.0-1.2] | 1.1 [1.0-1.2] | 1.1 [1.0-1.2] | 1.1 [1.0-1.2] | 1.1 [0.9-1.2] | 0.650 |
| Creatinine, time-to-peak, hours | 49 [17-78] | 50 [22-78] | 43 [13-74] | 50 [20-78] | 53 [19-78] | 0.125 |
| Hs-CRP, admission, ng/dL | 0.3 [0.1-0.5] | 0.2 [0.1-0.6] | 0.2 [0.1-0.5] | 0.2 [0.1-0.5] | 0.3 [0.1-0.6] | 0.142 |
| Hs-CRP, 6h, mg/dL | 0.3 [0.2-0.7] | 0.4 [0.1-0.6] | 0.3 [0.1-0.6] | 0.3 [0.2-0.6] | 0.4 [0.2-0.8] | **0.028** |
| Hs-CRP, 12h, mg/dL | 0.7 [0.4-1.3] | 0.7 [0.3-1.1] | 0.6 [0.3-1.0] | 0.8 [0.4-1.4] | 0.9 [0.5-1.6] | **<0.001** |
| Hs-CRP, 24h, mg/dL | 1.4 [0.7-2.7] | 1.1 [0.6-1.6] | 1.0 [0.6-1.7] | 1.5 [0.8-3.2] | 2.0 [1.1-3.6] | **<0.001** |
| Hs-CRP, 48h, mg/dL | 2.5 [1.2-4.4] | 2.2 [1.1-3.7] | 1.6 [0.9-2.7] | 2.9 [1.4-4.8] | 3.7 [2.2-6.8] | **<0.001** |
| Hs-CRP, 72h, mg/dL | 2.1 [1.1-3.9] | 1.3 [0.6-3.0] | 1.5 [0.7-2.7] | 2.4 [1.3-4.3] | 2.9 [1.7-5.8] | **<0.001** |
| Hs-CRP, peak, mg/dL | 2.7 [1.3-4.9] | 2.1 [0.8-4.3] | 1.7 [0.9-3.1] | 3.3 [1.6-5.3] | 4.2 [2.2-6.8] | **<0.001** |
| Hs-CRP, time-to-peak, hours | 47 [37-59] | 45 [36-55] | 46 [36-59] | 47 [40-60] | 47 [37-57] | 0.690 |
|  |  |  |  |  |  |  |
| Angiographic characteristics |  |  |  |  |  |  |
| Culprit lesion, n (%) |  |  |  |  |  | **<0.001** |
| RCA | 449 (41) | 19 (39) | 214 (50) | 115 (42) | 101 (28) |  |
| LAD | 478 (43) | 23 (47) | 153 (36) | 124 (45) | 178 (50) |  |
| LCX | 173 (16) | 6 (12) | 56 (13) | 34 (12) | 77 (21) |  |
| RI | 4 (<1) | 0 (0) | 0 (0) | 1 (<1) | 3 (1) |  |
| LM | 5 (<1) | 1 (2) | 4 (1) | - | - |  |
| Vessel disease, n (%) |  |  |  |  |  | 0.667 |
| Single-vessel | 619 (56) | 27 (55) | 245 (57) | 156 (57) | 191 (53) |  |
| Multi-vessel | 490 (44) | 22 (45) | 182 (43) | 118 (43) | 168 (47) |  |
| TIMI-flow 0 pre-PCI, n (%) | 657 (61) | 8 (19) | 198 (48) | 175 (66) | 276 (77) | **<0.001** |
| TIMI-flow 3 post-PCI, n (%) | 976 (91) | 42 (100) | 384 (94) | 232 (88) | 318 (89) | **0.005** |
|  |  |  |  |  |  |  |
| CMR Characteristics |  |  |  |  |  |  |
| Infarct size, % LVMM | 16 [8-26] | 0 [0-0] | 9 [4-15] | 19 [14-26] | 27 [18-36] | **<0.001** |
| LVEF, % | 50 [43-57] | 60 [53-64] | 53 [48-59] | 50 [42-55] | 45 [39-52] | **<0.001** |
| LVEDVi, mL/m^2^ | 80 [70-92] | 73 [63-87] | 77 [67-87] | 80 [70-91] | 85 [75-96] | **<0.001** |
| LVESVi, mL/m^2^ | 40 [31-49] | 30 [23-37] | 35 [28-44] | 41 [32-50] | 45 [38-55] | **<0.001** |
|  |  |  |  |  |  |  |
| Clinical Outcomes | | | | | | |
| MACE | 63 (7.0) | 0 (0) | 9 (2) | 7 (3.1) | 47 (15.7) | **<0.001** |
| All-cause death | 26 (2.9) | 0 (0) | 5 (1) | 2 (0.9) | 19 (6.3) | **<0.001** |

Abbreviations: CCS=Canadian Cardiovascular Society, TIMI=Thrombolysis in Myocardial Infarction, GRACE=Global Registry of Acute Coronary Events, Hs-cTnT=High-sensitivity cardiac troponin T, CK=Creatine kinase, NT-pro-BNP=N-terminal pro-B-type natriuretic peptide, Hs-CRP=High-sensitive C-reactive protein, RCA=Right coronary artery, LAD=Left anterior descending, LCX=Left circumflex artery, RI=Ramus intermedius, LM=Left main, PCI=Percutaneous coronary intervention, CMR=Cardiac magnetic resonance, LVMM=Left ventricular myocardial mass, LVEF=Left ventricular ejection fraction, MACE=Major adverse cardiovascular event, *data available for patients in the MARINA-STEMI study (n=600), ^†^data available for n=864 patients.

# Table S4: BASELINE CHARACTERISTICS ACCORDING TO CCS STAGES – CCS STAGE 1 DEFINED BY LGE <1% of LVMM

|  | Total population  (n=1,109) | CCS 1  (n=72, 7%) | CCS 2  (n=404, 36%) | CCS 3  (n=274, 25%) | CCS 4  (n=359, 32%) | p-value |
| --- | --- | --- | --- | --- | --- | --- |
| Patient characteristics |  |  |  |  |  |  |
| Age, years | 58 [51-68] | 59 [52-68] | 59 [51-68] | 58 [51-68] | 58 (52-68) | 0.982 |
| Female sex, n (%) | 242 (22) | 16 (22) | 95 (24) | 70 (26) | 61 (17) | 0.050 |
| Body mass index, kg/m^2^ | 27 [25-29] | 27 [25-30] | 27 [25-30] | 26 [25-29] | 27 [25-29] | 0.471 |
| Hypertension, n (%) | 503 (45) | 32 (44) | 175 (43) | 138 (50) | 158 (44) | 0.291 |
| Hyperlipidaemia, n (%) | 476 (43) | 38 (53) | 185 (46) | 108 (40) | 145 (40) | 0.093 |
| Diabetes mellitus, n (%) | 117 (11) | 12 (17) | 38 (9) | 26 (10) | 41 (11) | 0.261 |
| Smoker, n (%) | 608 (55) | 41 (57) | 224 (56) | 138 (51) | 205 (58) | 0.398 |
| TIMI-risk score | 3 [2-5] | 3 [1-4] | 3 [1-4] | 3 [2-5] | 3 [2-5] | **0.001** |
| GRACE 2.0 score^†^ | 108 [93-125] | 106 [94-122] | 104 [89-122] | 107 [93-123] | 111 [94-129] | 0.068 |
| Admission Killip-class |  |  |  |  |  | **<0.001** |
| 1 | 793 (72) | 59 (82) | 317 (79) | 203 (74) | 214 (60) |  |
| 2 | 281 (25) | 12 (17) | 83 (21) | 67 (25) | 119 (33) |  |
| 3 | 26 (2) | 0 (0) | 4 (21) | 3 (1) | 19 (5) |  |
| 4 | 9 (1) | 1 (1) | 0 (0) | 1 (<1) | 7 (2) |  |
| Acute kidney injury, n (%)* | 27 (2.4) | 1 (1.4) | 9 (2.2) | 8 (2.9) | 9 (2.5) | 0.873 |
| Total ischemic time, min | 179 [114-311] | 136 [95-267] | 174 [113-287] | 182 [115-310] | 200 [121-337] | **0.003** |
|  |  |  |  |  |  |  |
| Biomarkers characteristics |  |  |  |  |  |  |
| Hs-cTnT, admission, ng/L | 214 [45-1115] | 70 [28-207] | 133 [31-351] | 350 [63-1601] | 823 [78-2816] | **<0.001** |
| Hs-cTnT, 6h, ng/L | 3155 [1124-6628] | 356 [174-738] | 1474 [706-2920] | 3930 [1735-6884] | 6651 [4368-12038] | **<0.001** |
| Hs-cTnT, 12h, ng/L | 3936 [1764-6765] | 474 [182-1174] | 2160 [1162-3714] | 4576 [3044-6326] | 6830 [4377-9637] | **<0.001** |
| Hs-cTnT, 24h, ng/L | 3077 [1475-5177] | 381 [169-893] | 1679 [878-3190] | 3628 [2275-5087] | 4853 [3154-7106] | **<0.001** |
| Hs-cTnT, 48h, ng/L | 2317 [1260-3994] | 342 [140-782] | 1421 [811-2329] | 2832 [1838-3963] | 3559 [2313-5561] | **<0.001** |
| Hs-cTnT, 72h, ng/L | 2270 [1281-3896] | 411 [168-728] | 1429 [818-2310] | 2639 [1771-3786] | 3503 [2313-5335] | **<0.001** |
| Hs-cTnT, peak, ng/L | 4776 [2155-8076] | 660 [275-1190] | 2505 [1420-4671] | 5402 [3466-7533] | 8198 [5199-13816] | **<0.001** |
| Hs-cTnT, time-to-peak, hours | 11 [7-15] | 13 [8-36] | 13 [9-17] | 10 [6-16] | 8 [6-12] | **<0.001** |
| CK, admission, U/L | 305 [136-921] | 146 [76-223] | 211 [124-425] | 455 [158-1085] | 739 [215-1649] | **<0.001** |
| CK, 6h, U/L | 1576 [742-3177] | 225 [101-442] | 875 [472-1550] | 1727 [1002-3088] | 3288 [1843-4518] | **<0.001** |
| CK, 12h, U/L | 1618 [916-2786] | 255 [169-620] | 1014 [582-1707] | 1853 [1253-2853] | 2669 [1667-3865] | **<0.001** |
| CK, 24h, U/L | 1133 [608-1857] | 211 [143-687] | 786 [436-1254] | 1303 [861-1910] | 1732 [1072-2620] | **<0.001** |
| CK, 48h, U/L | 465 [267-769] | 127 [78-395] | 321 [190-570] | 520 [322-746] | 643 [395-1021] | **<0.001** |
| CK, 72h, U/L | 222 [136-371] | 93 [64-172] | 163 [109-286] | 241 [169-375] | 309 [209-498] | **<0.001** |
| CK, peak, U/L | 1913 [980-3508] | 293 [174-769] | 1164 [643-1865] | 2284 [1482-3345] | 3614 [2237-4736] | **<0.001** |
| CK, time-to-peak, hours | 8 [6-13] | 13 [8-19] | 11 [8-15] | 8 [6-13] | 6 [5-9] | **<0.001** |
| NT-pro-BNP, admission, ng/L | 170 [64-570] | 142 [50-455] | 155 [50-381] | 194 [66-804] | 183 [74-821] | **0.021** |
| NT-pro-BNP, 6h, ng/L | 355 [155-1007] | 218 [98-1039] | 286 [117-625] | 437 [162-1046] | 468 [208-1446] | **<0.001** |
| NT-pro-BNP, 12h, ng/L | 928 [498-1815] | 640 [292-1482] | 740 [394-1357] | 1034 [564-2013] | 1180 [701-2216] | **<0.001** |
| NT-pro-BNP, 24h, ng/L | 1302 [709-2332] | 610 [331-1557] | 976 [575-1762] | 1371 [750-2626] | 1677 [1015-2932] | **<0.001** |
| NT-pro-BNP, 48h, ng/L | 1132 [607-2095] | 561 [306-1080] | 904 [459-1602] | 1190 [656-2404] | 1501 [920-2862] | **<0.001** |
| NT-pro-BNP, 72h, ng/L | 1009 [469-1797] | 413 [258-953] | 681 [328-1411] | 1045 [513-2035] | 1433 [803-2627] | **<0.001** |
| NT-pro-BNP, peak, ng/L | 1453 [758-2716] | 702 [397-1801] | 1106 [634-1908] | 1470 [838-3219] | 2107 [1179-3651] | **<0.001** |
| NT-pro-BNP, time-to-peak, hours | 23 [17-44] | 20 [14-38] | 21 [17-37] | 24 [16-44] | 25 [19-45] | **0.026** |
| Creatinine, admission, U/L | 1.0 [0.8-1.1] | 1.0 [0.9-1.1] | 0.9 [0.8-1.1] | 1.0 [0.8-1.1] | 0.9 [0.8-1.1] | 0.730 |
| Creatinine, 6h, mg/dL | 0.9 [0.8-1.0] | 0.9 [0.8-1.0] | 0.9 [0.8-1.0] | 0.9 [0.8-1.0] | 0.9 [0.8-1.0] | 0.683 |
| Creatinine, 12h, mg/dL | 0.9 [0.8-1.0] | 1.0 [0.9-1.0] | 0.9 [0.8-1.0] | 0.9 [0.8-1.0] | 0.9 [0.8-1.1] | 0.571 |
| Creatinine, 24h, mg/dL | 0.9 [0.8-1.1] | 1.0 [0.9-1.1] | 0.9 [0.8-1.1] | 1.0 [0.8-1.1] | 0.9 [0.8-1.1] | 0.766 |
| Creatinine, 48h, mg/dL | 1.0 [0.9-1.1] | 1.0 [0.9-1.1] | 1.0 [0.9-1.1] | 1.0 [0.9-1.1] | 1.0 [0.9-1.1] | 0.865 |
| Creatinine, 72h, mg/dL | 1.0 [0.9-1.1] | 1.0 [1.0-1.1] | 1.0 [0.8-1.1] | 1.0 [0.9-1.1] | 1.0 [0.9-1.1] | 0.396 |
| Creatinine, peak, mg/dL | 1.1 [1.0-1.2] | 1.1 [1.0-1.2] | 1.1 [0.9-1.2] | 1.1 [1.0-1.2] | 1.1 [0.9-1.2] | 0.382 |
| Creatinine, time-to-peak, hours | 49 [17-78] | 49 [23-70] | 44 [13-75] | 50 [20-78] | 53 [19-78] | 0.175 |
| Hs-CRP, admission, ng/dL | 0.3 [0.1-0.5] | 0.2 [0.1-0.4] | 0.2 [0.1-0.5] | 0.2 [0.1-0.5] | 0.3 [0.1-0.6] | 0.109 |
| Hs-CRP, 6h, mg/dL | 0.3 [0.2-0.7] | 0.3 [0.1-0.6] | 0.3 [0.1-0.6] | 0.3 [0.2-0.6] | 0.4 [0.2-0.8] | **0.024** |
| Hs-CRP, 12h, mg/dL | 0.7 [0.4-1.3] | 0.4 [0.3-1.0] | 0.6 [0.3-1.0] | 0.8 [0.4-1.4] | 0.9 [0.5-1.6] | **<0.001** |
| Hs-CRP, 24h, mg/dL | 1.4 [0.7-2.7] | 1.0 [0.4-1.6] | 1.1 [0.6-1.7] | 1.5 [0.8-3.2] | 2.0 [1.1-3.6] | **<0.001** |
| Hs-CRP, 48h, mg/dL | 2.5 [1.2-4.4] | 1.8 [1.0-3.3] | 1.6 [0.9-2.7] | 2.9 [1.4-4.8] | 3.7 [2.2-6.8] | **<0.001** |
| Hs-CRP, 72h, mg/dL | 2.1 [1.1-3.9] | 1.2 [0.6-2.6] | 1.5 [0.7-2.8] | 2.4 [1.3-4.3] | 2.9 [1.7-5.8] | **<0.001** |
| Hs-CRP, peak, mg/dL | 2.7 [1.3-4.9] | 1.9 [0.7-3.5] | 1.8 [0.9-3.1] | 3.3 [1.6-5.3] | 4.2 [2.2-6.8] | **<0.001** |
| Hs-CRP, time-to-peak, hours | 47 [37-59] | 33 [46-59] | 46 [36-59] | 47 [40-60] | 47 [37-57] | **0.792** |
|  |  |  |  |  |  |  |
| Angiographic characteristics |  |  |  |  |  |  |
| Culprit lesion, n (%) |  |  |  |  |  | **<0.001** |
| RCA | 449 (41) | 30 (42) | 203 (50) | 115 (42) | 101 (28) |  |
| LAD | 478 (43) | 32 (44) | 144 (36) | 124 (45) | 178 (50) |  |
| LCX | 173 (16) | 9 (13) | 53 (13) | 34 (12) | 77 (21) |  |
| RI | 4 (<1) | 0 (0) | 0 (0) | 1 (<1) | 3 (1) |  |
| LM | 5 (<1) | 1 (1) | 1 (1) | - | - |  |
| Vessel disease, n (%) |  |  |  |  |  | 0.358 |
| Single-vessel | 619 (56) | 36 (50) | 236 (58) | 156 (57) | 191 (53) |  |
| Multi-vessel | 490 (44) | 36 (50) | 168 (42) | 118 (43) | 168 (47) |  |
| TIMI-flow 0 pre-PCI, n (%) | 657 (61) | 14 (22) | 192 (50) | 175 (66) | 276 (77) | **<0.001** |
| TIMI-flow 3 post-PCI, n (%) | 976 (91) | 64 (99) | 362 (94) | 232 (88) | 318 (89) | **0.005** |
|  |  |  |  |  |  |  |
| CMR Characteristics |  |  |  |  |  |  |
| Infarct size, % LVMM | 16 [8-26] | 0 [0-0.3] | 10 [5-15] | 19 [14-26] | 27 [18-36] | **<0.001** |
| LVEF, % | 50 [43-57] | 60 [54-64] | 53 [48-59] | 50 [42-55] | 45 [39-52] | **<0.001** |
| LVEDVi, mL/m^2^ | 80 [70-92] | 72 [61-86] | 77 [67-88] | 80 [70-91] | 85 [75-96] | **<0.001** |
| LVESVi, mL/m^2^ | 40 [31-49] | 29 [23-35] | 30 [28-45] | 41 [32-50] | 45 [38-55] | **<0.001** |
|  |  |  |  |  |  |  |
| Clinical Outcomes | | | | | | |
| MACE | 63 (7.0) | 0 (0) | 9 (2.2) | 7 (3.1) | 47 (15.7) | **<0.001** |
| All-cause death | 26 (2.9) | 0 (0) | 5 (1.2) | 2 (0.9) | 19 (6.3) | **<0.001** |

Abbreviations: CCS=Canadian Cardiovascular Society, TIMI=Thrombolysis in Myocardial Infarction, GRACE=Global Registry of Acute Coronary Events, Hs-cTnT=High-sensitivity cardiac troponin T, CK=Creatine kinase, NT-pro-BNP=N-terminal pro-B-type natriuretic peptide, Hs-CRP=High-sensitive C-reactive protein, RCA=Right coronary artery, LAD=Left anterior descending, LCX=Left circumflex artery, RI=Ramus intermedius, LM=Left main, PCI=Percutaneous coronary intervention, CMR=Cardiac magnetic resonance, LVMM=Left ventricular myocardial mass, LVEF=Left ventricular ejection fraction, MACE=Major adverse cardiovascular event, *data available for patients in the MARINA-STEMI study (n=600), ^†^data available for n=864 patients.

**TABLE S5: COX REGRESSION ANALYSIS FOR THE PREDICTION OF MACE**

|  | Univariable | | Multivariable | |
| --- | --- | --- | --- | --- |
|  | HR [95% CI] | p-value | HR [95% CI] | p-value |
| Female sex | 0.85 [0.71-1.01] | 0.064 | - | - |
| Body mass index | 0.97 [0.79-1.17] | 0.726 | - | - |
| Hyperlipidaemia | 1.19 [0.98-1.45] | 0.082 | - | - |
| Smoking | 1.02 [0.84-1.25] | 0.82 | - | **-** |
| Acute kidney injury | 0.53 [0.12-2.26] | 0.390 | - | - |
| Culprit lesion | 1.09 [0.90-1.31] | 0.395 | - | - |
| Multivessel disease | 1.48 [1.21-1.81] | **<0.001** | 1.87 [1.34-2.59] | **<0.001** |
| TIMI-flow 0 pre-PCI | 1.46 [1.16-1.83] | **0.001** | - | **-** |
| TIMI-flow 3 post-PCI | 0.84 [0.71-0.99] | **0.046** | - | - |
| LVEF | 0.53 [0.44-0.64] | **<0.001** | - | - |
| LVEDVi | 1.29 [1.06-1.57] | **0.013** | - | - |
| LVESVi | 1.60 [1.35-1.89] | **<0.001** | - | - |
| TIMI-risk score | 2.27 [1.91-2.70] | **<0.001** | 2.28 [1.70-3.08] | **<0.001** |
| GRACE 2.0 score^†^ | 2.25 [1.86-2.73] | **<0.001** | - | **-** |
| CCS stages | 2.18 [1.70-2.78] | **<0.001** | 2.45 [1.59-3.77] | **<0.001** |

Abbreviations: HR=Hazard ratio, CI=Confidence interval, TIMI=Thrombolysis in Myocardial Infarction, PCI=Percutaneous coronary intervention, LVEF=Left ventricular ejection fraction, LVEDVi=Left ventricular end diastolic volume index, LVESVi=Left ventricular end systolic volume index, GRACE=Global Registry of Acute Coronary Events, CCS=Canadian Cardiovascular Society, ^†^data available for n=864 patients.
